# Surface Planarization‐Epitaxial Growth Enables Uniform 2D/3D Heterojunctions for Efficient and Stable Perovskite Solar Modules

**DOI:** 10.1002/advs.202407380

**Published:** 2024-11-03

**Authors:** Dongxu Lin, Jun Fang, Sibo Li, Zhenye Zhan, Huan Li, Xin Wang, Guanshui Xie, Daozeng Wang, Nuanshan Huang, Haichen Peng, Weiguang Xie, Luis K. Ono, Yabing Qi, Longbin Qiu

**Affiliations:** ^1^ Shenzhen Key Laboratory of Intelligent Robotics and Flexible Manufacturing Systems Department of Mechanical and Energy Engineering SUSTech Energy Institute for Carbon Neutrality Southern University of Science and Technology Shenzhen 518055 P. R. China; ^2^ College of Physics & Optoelectronic Engineering Jinan University Guangzhou Guangdong 510632 P. R. China; ^3^ Energy Materials and Surface Sciences Unit (EMSSU) Okinawa Institute of Science and Technology Graduate University (OIST) 1919‐1 Tancha, Onna‐son Kunigami‐gun Okinawa 904‐0495 Japan; ^4^ Global Institute of Future Technology Shanghai Jiao Tong University Shanghai 200240 P. R. China

**Keywords:** 2D/3D heterojunction perovskites, modules, stability, surface planarization‐epitaxial growth

## Abstract

Two‐dimensional/three‐dimensional (2D/3D) halide perovskite heterojunctions are widely used to improve the efficiency and stability of perovskite solar cells. However, interfacial defects between the 2D and 3D perovskites and the poor coverage of the 2D capping layer still hinder long‐term stability and homogeneous charge extraction. Herein, a surface planarization strategy on 3D perovskite is developed that enables an epitaxial growth of uniform 2D/3D perovskite heterojunction via a vapor‐assisted process. The homogeneous charge extraction and suppression of interfacial nonradiative recombination is achieved by forming a uniform 2D/3D interface. As a result, a stabilized power output efficiency of 25.97% is achieved by using a 3D perovskite composition with a bandgap of 1.55 eV. To demonstrate the universality of the strategy applied for different perovskites, the champion device based on a 1.57 eV bandgap 3D perovskite results in an efficiency of 25.31% with a record fill factor of 87.6%. Additionally, perovskite solar modules achieve a designated area (24.04 cm^2^) certified efficiency of 20.75% with a high fill factor of 80.0%. Importantly, the encapsulated uniform 2D/3D modules retain 96.9% of the initial efficiency after 1246 h operational tracking under 65 °C (ISOS‐L‐3 protocol) and 91.1% after 862 h under the ISOS‐O‐1 protocol.

## Graphical abstract

By employing a surface planarization strategy, a uniform and dense p‐i‐n 2D/3D heterojunction is successfully developed. This approach has enabled planarized 2D/3D PSCs and modules to achieve remarkable efficiencies of 26.02% for a 0.1 cm^2^ area and 23.06% for a 22.8 cm^2^ area, primarily by suppressing interface nonradiative recombination. Additionally, these devices exhibit exceptional operational stability under accelerated testing protocols.

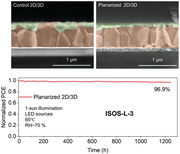

## 1 Introduction

Metal halide perovskite solar cells (PSCs) have garnered significant attention owing to their low fabrication costs and exceptional optoelectronic properties.^[^ ^1^, ^2^, ^3^, ^4^ ^]^ Inverted PSCs, with their excellent stability and low fabrication costs, have recently achieved impressive PCEs by incorporating self‐assembled monolayers (SAMs) as hole‐selective layers.^[^ ^5^, ^6^, ^7^, ^8^ ^]^ To date, the certified power conversion efficiency (PCE) of small‐area single‐junction PSCs has reached 26.7%. Despite successful application of SAMs in fabricating high‐efficiency p‐i‐n PSCs, there remains a gap in PCE between p‐i‐n and n‐i‐p perovskite solar modules (PSMs). Currently, certified efficiencies of n‐i‐p PSMs have reached 23.30% with an aperture area of 27.22 cm^2^,^[^ ^9^ ^]^ surpassing those of p‐i‐n PSMs (certified 21.8% for an aperture area of 26.9 cm^2^). One of the reasons is related to the parasitic absorption and/or energy alignment mismatch of poly[bis(4‐phenyl)(2,4,6‐trimethylphenyl]amine (PTAA) employed as the hole transport layer on the glass side of the p‐i‐n structure.^[^ ^10^, ^11^ ^]^ Further development of SAMs‐based PSMs is pivotal in bridging the efficiency gap between n‐i‐p and p‐i‐n PSMs (Table S1, Supporting Information). In addition, developing high‐performance and long‐term stable PSMs for commercial deployment remains a significant challenge, due to inhomogeneity of interfacial charge extraction layers in large‐area thin films and the inherent instability of the perovskite absorber layer.^[^ ^12^, ^13^, ^14^, ^15^ ^]^

Two‐dimensional halide perovskites, known for their excellent stability, are commonly used to enhance the performance and stability of 3D PSCs by creating a 2D/3D perovskite heterojunction.^[^ ^16^, ^17^, ^18^, ^19^, ^20^ ^]^ Most of the current 2D/3D perovskite heterojunctions are fabricated through a solution cation exchange strategy, where a large organic cation salt solution is spin‐coated on top of 3D perovskites.^[^ ^21^, ^22^ ^]^ However, the solution cation exchanged 2D/3D perovskite will form a graded junction due to cation interdiffusion.^[^ ^23^, ^24^ ^]^ This graded junction interface is considered to be a cause leading to the decomposition pathway for 2D/3D perovskites, posing a detrimental effect on the stability of heterojunction solar cells.^[^ ^25^, ^26^ ^]^ Furthermore, the cation exchange strategy results in a multi‐n‐valued 2D perovskite capping layer, leading to inhomogeneous charge extraction in large‐area solar modules.^[^ ^27^ ^]^ Constructing a well‐defined interface 2D/3D perovskite heterojunction can maximize the stability advantages. Using a solvent for 2D perovskite fabrication that does not disturb the underneath 3D perovskite layer enables the creation of 2D/3D perovskite heterojunctions with sharp interfaces via the spin‐coating method.^[^ ^18^, ^28^ ^]^ The vapor‐assisted growth strategy is alternative effective method for fabricating uniform and sharp 2D/3D interfaces, leading to significant enhancements in the stability of PSCs and PSMs.^[^ ^17^, ^29^, ^30^ ^]^ The above studies demonstrate that constructing a sharp interfacial 2D/3D perovskite heterojunction through vapor‐assisted growth is crucial for achieving high‐efficiency and stable inverted PSCs and PSMs.

However, the required thickness of the 2D perovskite capping layer varies based on the solar cell architecture.^[^ ^18^ ^]^ Typically, the thickness of the 2D perovskite capping layer should be controlled to less than tens of nanometers in the p‐i‐n architecture,^[^ ^29^, ^31^ ^]^ which remains a challenge for achieving uniform coverage in such thin layers. Considering the significant volume expansion during the transformation from PbI_2_ to 2D perovskites, the thickness of the vapor deposited PbI_2_ precursor thin film should be a few nanometers. This poses a challenge in achieving uniform 2D perovskite capping layers on the surface of 3D perovskites. Despite the extensive research on 2D/3D perovskite heterojunction, less attention has been given to the effects of 3D perovskite surface states.^[^ ^28^ ^]^ The surface states of 3D perovskites, including surface energies and lattice parameters, determine the growth mode of vapor‐deposited PbI_2_ films. Moreover, due to the physical contact between vapor‐deposited PbI_2_ and 3D perovskites during the vapor‐assisted growth of 2D/3D perovskite heterojunction, the surface defects of 3D perovskite will remain and act as interface defects between 2D/3D perovskite heterojunction, leading to an increase in carrier recombination.^[^ ^17^ ^]^

Herein, we make use of surface planarization‐epitaxial growth to fabricate uniform and dense p‐i‐n 2D/3D heterojunction PSCs with a low interfacial defect density. Our results demonstrate that the surface planarization process induces the formation of a Pb‐rich surface on the 3D perovskite, enhancing lattice matching during PbI_2_ deposition. It also reduces surface roughness and increases surface energy, promoting the Frank–van der Merwe growth (layer‐by‐layer growth) of vapor‐deposited PbI_2_ and enabling uniform and dense 2D perovskite capping layer formation. Moreover, the surface planarization‐epitaxial growth strategy reduces the surface defect concentration of the 3D perovskite, suppressing interface recombination between 2D and 3D perovskites. Finally, the planarized p‐i‐n 2D/3D PSCs achieve a champion PCE of 25.31% for 1.57 eV bandgap Cs_0.05_MA_0.1_FA_0.85_PbI_2.9_Br_0.1_ and 26.02% for 1.55 eV Cs_0.05_MA_0.1_FA_0.85_PbI_3_, which represents the highest efficiency for 2D/3D heterojunction PSCs (Figure S1, Supporting Information). Moreover, owing to the uniform 2D perovskite capping layer, the planarized 2D/3D heterojunctions demonstrate a homogeneous charge extraction ability in large areas, achieving an active area (22.8 cm^2^) PCE of 23.06% in PSM. The designated area (24.04 cm^2^) efficiency is measured to be 21.4% with a high fill factor of 81.0%. The fill factor is ranked high among the certified PSMs (Table S1, Supporting Information), attributing to the reduced trap states and nonradiative recombination. In addition, the designated area efficiency of PSM is certified to be 20.75% by the National PV Industry Measurement and Testing Center. Notably, the encapsulated PSMs maintain 96.9% after 1246 h under one‐sun illumination and maximum power point (MPP) tracking at 65 °C (ISOS‐L‐3 protocol).

## 2 Results and Discussion

**Figure 1:**
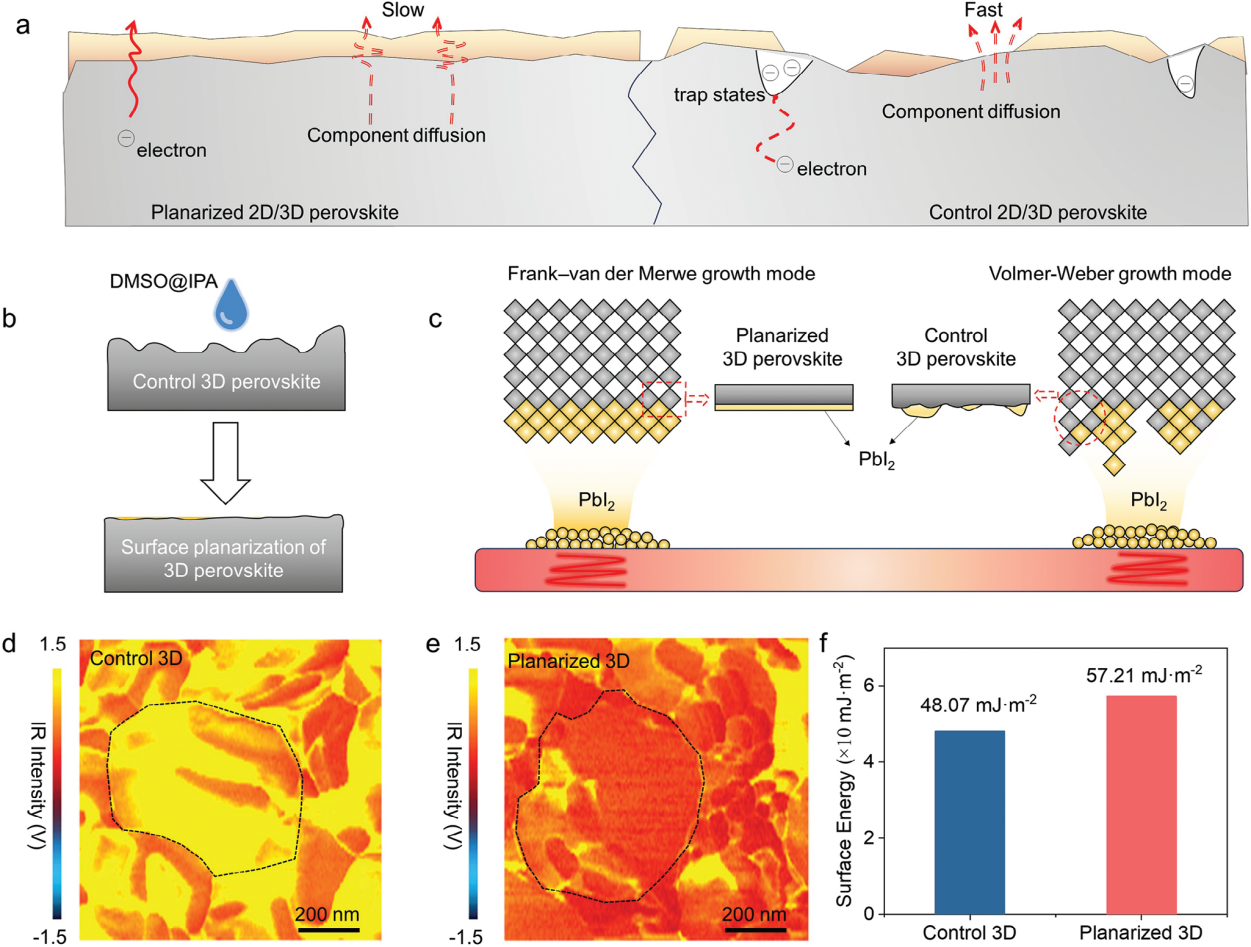
Scheme and effect of planarized 3D perovskites. a) Illustration of the benefits of planarized 2D/3D perovskites heterojunction. The surface planarization of 3D perovskites helps form a uniform 2D capping layer and reduce interfacial defects, which can slow down the outward diffusion of the components from perovskite, suppress interfacial nonradiative recombination, and improve the homogeneity of charge extraction. b) Illustration of the surface planarization. The DMSO@IPA can planarize the surface of 3D perovskite. c) Illustration of the growth model of PbI_2_ deposited on planarized and control 3D perovskites. The planarized 3D perovskite, with a higher surface energy and better lattice matching with PbI_2_, can form a uniform PbI_2_ layer. In contrast, the vapor‐deposited PbI_2_ on the surface of the control 3D perovskite is discontinuous. d,e) AFM‐IR images of (d) control 3D perovskite and (e) planarized 3D perovskite. f) The surface energy of control and planarized 3D perovskites.

It is found that the surface planarization‐epitaxial growth strategy can form a homogeneous and uniform 2D capping layer on 3D perovskite, preventing the escape of components and irreversible decomposition.^[^ ^32^ ^]^ Meanwhile, planarization eliminates interfacial defects between 2D and 3D perovskites, effectively suppressing interfacial nonradiative recombination, as shown in **Figure** **1**a. A mixed solvent composed of isopropyl alcohol (IPA) and dimethyl sulfoxide (DMSO) with a volume ratio of 200:1 was employed as a planarization agent to reconstruct the surface of 3D perovskites, as shown in Figure 1b. Incorporating a small amount of polar aprotic DMSO solvent in the planarization agent can improve the solubility of 3D perovskites.^[^ ^33^ ^]^ Planarized and lattice‐matched surfaces enable the Frank–van der Merwe epitaxial growth of PbI_2_ during vapor deposition, a prerequisite for forming homogeneous and uniform 2D capping layers on 3D perovskite, as illustrated in Figure 1c. Figure S2a,b (Supporting Information) displays optical images of 3D perovskites before and after planarization. The control 3D perovskite film (before planarized) exhibits a rough texture and a greyish‐black surface, markedly differing from the planarized 3D perovskite film, which has a shiny black mirror‐like appearance. This stark contrast indicates a significant alteration in the surface state attributable to the surface planarization process. We then employed grazing incident X‐ray diffraction (GIXRD) at a small incidence angle to examine the changes in surface components after surface planarization. As shown in Figure S3 (Supporting Information), the (001) peak of PbI_2_ is observed on the top of the planarized 3D perovskite, while in the control 3D perovskite, there is no corresponding signal at the incidence angle of 0.1°. This result indicates the formation of a PbI_2_‐rich surface after planarization, which is expected to enhance lattice matching during PbI_2_ vapor deposition. Additionally, the phase changes in the 3D perovskite induced by the planarization agent have been excluded (Figure S4, Supporting Information).

We further conducted scanning electron microscopy (SEM) measurements to study the surface differences and to examine the morphological changes induced by surface planarization (Figure S5, Supporting Information). The control 3D perovskite film in Figure S5a (Supporting Information) exhibits a typical perovskite morphology with well‐defined grain boundaries, and its cross‐sectional SEM in Figure S5c (Supporting Information) shows a relatively rough surface. In contrast, the planarized 3D perovskite in Figure S5b (Supporting Information) shows a substantial presence of bright crystals, indexed as PbI_2_, predominantly located around the grain boundaries. This PbI_2_ filling at grain boundaries effectively planarizes the surface as shown in Figure S5d (Supporting Information), thereby reducing the surface roughness, as further confirmed by atomic force microscopy (AFM) in Figure S6 (Supporting Information). Meanwhile, we found that surface planarization effectively reduces surface trap states, as shown in Figure S7 (Supporting Information). It is well known that DMSO is a common perovskite solvent that can easily dissolve perovskites and their precursors. However, when DMSO is diluted in IPA at a volume ratio of 1:200, forming the planarization agent, it can only dissolve FAI but not PbI_2_, as demonstrated in Figure S8 (Supporting Information). This suggests that the amorphous phases and regions of poor crystallinity on the perovskite surface can be degraded into PbI_2_ by the planarization agent. Figure S9 (Supporting Information) shows the perovskite films with and without the MACl additive immersed in the planarization agent for 5 h. Since the perovskite film without MACl exhibits poor crystallinity and contains a large amount of amorphous phase, it easily degrades into PbI_2_ after immersion in the planarization agent. The UV–vis spectra show a significant I_3_^−^ peak only in the soaking solution of the perovskite film without MACl. This indicates that the planarization agent effectively dissolves the amorphous and poorly crystalline perovskite regions. Cross‐sectional high‐resolution transmission electron microscopy (HRTEM) was used to directly observe the elimination of the surface amorphous phase by the planarization agent. As shown in Figure S10 (Supporting Information), the control 3D perovskites exhibit a noticeable amorphous phase on the surface, while the planarized 3D perovskites display good surface crystallinity. Both UV–vis and HRTEM analyses demonstrate that the planarization agent can reduce trap states by eliminating the amorphous phase in perovskite films, which are commonly found at the surface and grain boundaries of solution‐processed polycrystalline perovskite films. Additionally, since the planarization agent does not dissolve PbI_2_, the PbI_2_ formed by decomposition fills the valleys on the perovskite film surface due to centrifugal force.

We further employed atomic force microscopy‐infrared spectroscopy (AFM‐IR) measurements to investigate the grain interiors of perovskites. We map the AFM‐IR imaging at 1712 cm^−1^, corresponding to the signals of FA^+^ cations.^[^ ^34^ ^]^ As depicted in Figure 1d,e and Figure S11 (Supporting Information), the grain interiors (indicated by the black dashed contours) of the control 3D perovskite exhibit strong IR intensity, suggesting a higher concentration of FA^+^ cations. Conversely, the planarized 3D perovskite displays lower signals of FA^+^ cations. These results suggest planarization leads to an ultrathin PbI_2_ layer covering the grain interiors.

The planarization process applied to 3D perovskite can also significantly affect the surface energy, as evidenced in Figure 1f. The surface energy was obtained using contact angle measurements (Figure S12, Supporting Information). The analysis employed the Owens‐Wendt method, accounting for polar and dispersive surface energy components, as provided in Note S1 (Supporting Information). The surface energy of the planarized 3D perovskite film markedly increased from 48.07 to 57.21 mJ m^−^^2^ as shown in Table S2 (Supporting Information), which can be attributed to the alterations in surface roughness and composition induced by the surface planarization process. The morphology of the vacuum‐deposited thin film depends on the properties of substrate.^[^ ^35^, ^36^ ^]^ The Frank–van der Merwe growth mode, known as layer‐by‐layer film growth, is extensively researched for its critical role in producing smooth and uniform thin films.^[^ ^37^ ^]^ Surface roughness, surface energy, and lattice parameters of the substrate are key factors for achieving Frank–van der Merwe growth in vacuum‐deposited thin films. Ideally, a smoother substrate with minimal roughness is preferred as it facilitates uniform nucleation and growth of the film.^[^ ^35^ ^]^ Besides, higher surface energy typically promotes better wetting and adhesion, enabling the formation of continuous and uniform layers.^[^ ^38^ ^]^ A proper lattice matching between the substrate and deposited film helps reduce strain and prevent the formation of defects, thereby ensuring the smooth growth of uniform layers.^[^ ^39^, ^40^ ^]^

**Figure 2:**
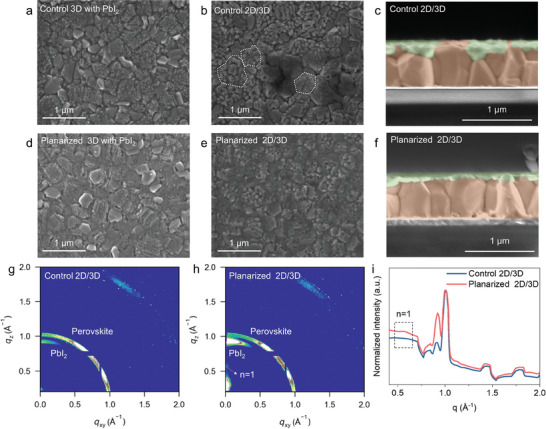
Epitaxy vapor‐assisted growth of uniform 2D/3D perovskites. Top surface SEM images of (a) control 3D perovskite with vapor‐deposited PbI_2_ and (b) control 2D/3D perovskite. c) A cross‐sectional SEM image of the planarized 2D/3D perovskite. Top surface SEM images of (d) planarized 3D perovskite with vapor‐deposited PbI_2_ and (e) planarized 2D/3D perovskite. f) A cross‐sectional SEM image of the planarized 2D/3D perovskite. GIWAXS patterns of (g) the control 2D/3D and (h) the planarized 2D/3D perovskite films. i) Radial intensity profiles averaged over the entire 2D GIWAXS image.

The alteration in the surface properties of surface planarized 3D perovskites, encompassing changes in surface energy, roughness, and composition, facilitates the formation of a continuous and uniform ultrathin PbI_2_ layer, following the Frank–van der Merwe growth mode, as demonstrated in Figure S13 (Supporting Information). When 4 nm PbI_2_ deposited on perovskite films, for the control 3D perovskite, SEM images in Figure S14a,b (Supporting Information) reveal that PbI_2_ cannot fully cover the perovskite surface. As highlighted in the red circle, the growth of vapor‐deposited PbI_2_ aggregates leads to the exposure of 3D perovskite. However, the planarized 3D perovskite demonstrates effective coverage with 4 nm PbI_2_, as shown in Figure S14c,d (Supporting Information). **Figure** **2**a,d display the surface morphology of 4 nm PbI_2_ deposited on both control and planarized 3D perovskite surfaces. PbI_2_ exhibits a discontinuous and uneven outward growth on the control surface. While on the planarized surface, it appears flat and fully covers the substrate without pinholes. To further verify the full coverage of the 4 nm PbI_2_ deposited on planarized 3D perovskites, we adjusted the SEM sample holder angle to capture both surface and cross‐sectional morphology simultaneously at a magnification of 10000. As shown in Figure S15 (Supporting Information), the surface exhibited uniform morphology. Additionally, cathodoluminescence (CL) mapping was used to record PbI_2_ emission within the spectral window of 530 to 590 nm, as shown in Figure S16 (Supporting Information). Both the morphology and CL mapping confirmed that the 4 nm PbI_2_ had good coverage on the planarized perovskite. The conversion of PbI_2_ into 2D perovskite is then achieved by spin‐coating PEABr onto these PbI_2_/3D‐perovskite films. Figure 2b shows the formation of a discontinuous 2D capping layer on control 3D perovskite, further elucidated by the cross‐sectional SEM image in Figure 2c. In contrast, Figure 2e and its cross‐sectional SEM image (Figure 2f) depict a homogeneous and compact 2D capping layer with a thickness of ≈ 20 nm on planarized 3D perovskite. The SEM findings underscore the significance of the surface planarization process in 2D/3D perovskite heterojunction fabrication. Achieving a uniformly covered PbI_2_ precursor film is crucial, as it facilitates the formation of a homogeneous and uniform 2D/3D perovskite heterostructure.

The crystal orientation of the control and planarized 2D/3D perovskite films was investigated using the grazing‐incidence wide‐angle X‐ray scattering (GIWAXS) technique. The scattering patterns of these perovskite films are depicted in Figure 2g,h, while their corresponding radial intensity profiles, averaged over the entire images, are presented in Figure 2i. Both control and planarized 2D/3D perovskite films exhibit a diffraction ring at a scattering vector of q = 1 Å⁻¹, indicating the random orientation of 3D perovskite.^[^ ^41^ ^]^ In the control 2D/3D perovskite film, no distinct 2D perovskite component was detected, which means that the PEABr in the control 3D perovskite only forms a small quantity of multi‐n‐value 2D components. As the PbI_2_ layer on the control 3D perovskite is discontinuous, the spin‐coated PEABr on the surface will react with both PbI_2_ and bottom 3D perovskite, leading to the formation of these multi‐n‐value 2D components. Conversely, for the planarized 2D/3D perovskite films, distinct diffraction peaks are observed at a scattering vector of q = 0.56 Å⁻¹ (Figure 2i), which are identified as belonging to the n = 1 2D perovskite.^[^ ^42^, ^43^ ^]^ This indicates the formation of a single n‐value 2D capping layer, attributed to the continuous and homogeneous PbI_2_ layer formed on the planarized 3D perovskite. To further confirm the formation of a single n‐value 2D capping layer on planarized 3D perovskite, photoluminescence (PL) spectra were recorded from the perovskite side using 405 nm laser excitation. As shown in Figure S17a (Supporting Information), the planarized 2D/3D perovskite exhibited an n = 1 2D perovskite emission peak, while the control 2D/3D perovskite showed multiple emission peaks corresponding to various n values (n = 1, 2, and 3) 2D perovskite. Additionally, no distinct 2D excitonic absorption peak was observed in the UV–vis spectrum (Figure S17b, Supporting Information), indicating that the content of 2D perovskite is minimal. The GIWAXS and PL results confirm that the surface planarization process facilitates the formation of a homogeneous 2D capping layer with a single n‐value, highlighting its effectiveness in enhancing the structural uniformity of 2D/3D perovskite films.

**Figure 3:**
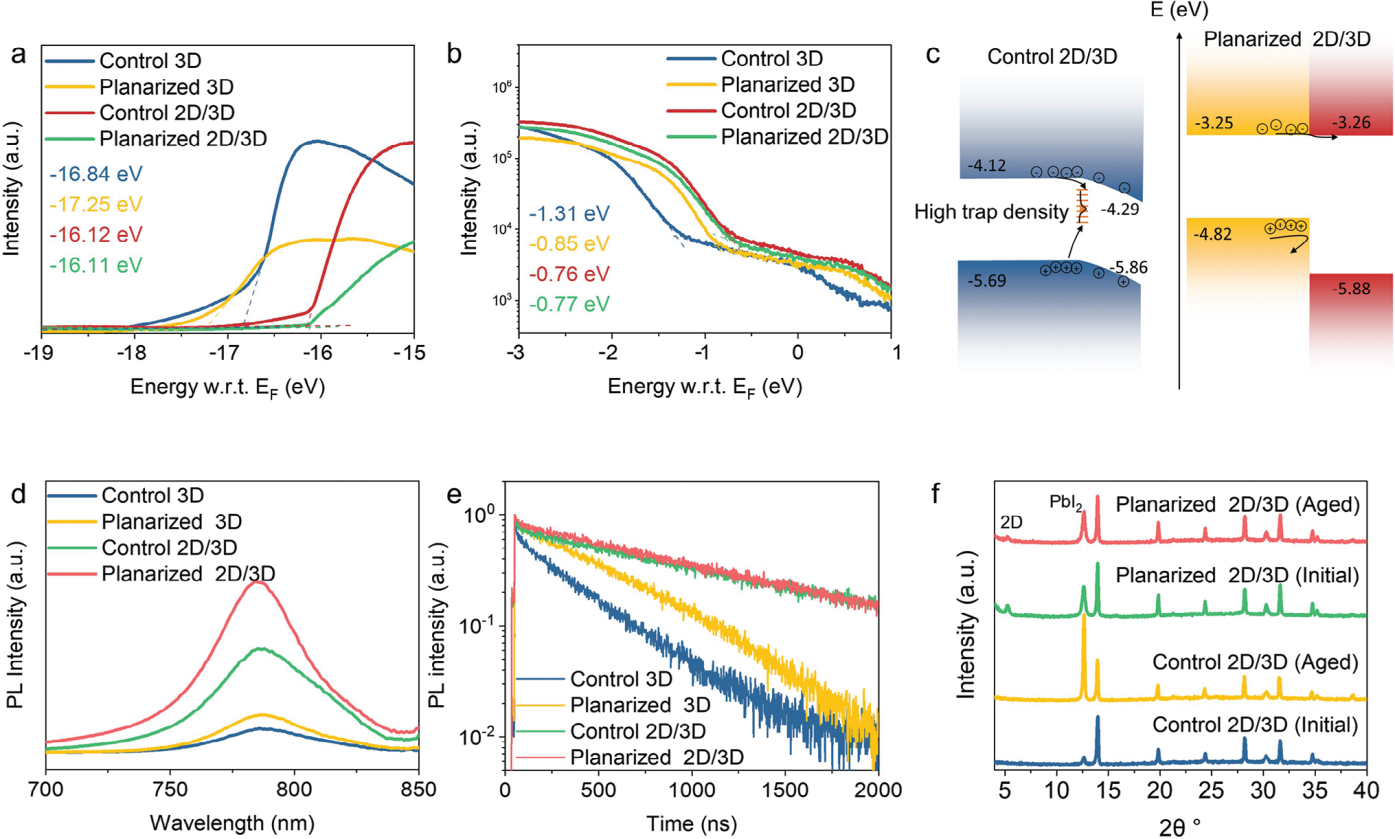
Carrier transport performance of planarization‐epitaxial growth uniform 2D/3D perovskite heterojunction. UPS spectra: a) the secondary electron onset region and b) the valence band region for control 3D, planarized 3D, control 2D/3D, and planarized 2D/3D perovskite films. c) Schematic of energy diagram for the control 2D/3D and planarized 2D/3D perovskite. The orange lines denote the surface trap state in control 3D perovskite, and the black arrows denote the nonradiative recombination pathways and the directions of carrier drift. The interface between control 3D and 2D forms a type‐I band alignment, whereas the interface between planarized 3D and 2D forms a type‐II band alignment. d) Steady‐state PL spectra and (e) TRPL decay curves of control 3D, planarized 3D, control 2D/3D, and planarized 2D/3D perovskite films deposited on glass substrates. f) XRD patterns of control 2D/3D and planarized 2D/3D perovskites before and after aging at 100 °C for 120 min.

We then conducted a systematic ultraviolet photoemission spectroscopy (UPS) study to demonstrate the effects of surface planarization on the energy‐level alignment at the 2D/3D interface. The bandgaps of the 3D and n = 1 2D perovskites were determined to be 1.57 and 2.62 eV, respectively, based on the Tauc plot analysis (Figure S18, Supporting Information). As depicted in **Figure** **3**a,b, the UPS spectra provided the work function and valence band onset values of 4.38 eV and 1.31 eV for the control 3D perovskite, 3.97 and 0.85 eV for the planarized 3D perovskite, 5.10 and 0.76 eV for the control 2D/3D perovskite, and 5.11 and 0.77 eV for the planarized 2D/3D perovskite. Since the 2D perovskites on the control 3D perovskite consist of multiple n‐value components and exhibit a discontinuous morphology, the energy band alignment should be described similarly to those surface passivation using large ammonium cations literatures. In contrast, for the planarized 2D/3D perovskite, where SEM images reveal a well‐defined bilayer structure and a uniform single n‐value 2D capping layer, the energy band alignment can be constructed using the individual energy bands of 2D and 3D perovskites. The energy band alignment of the perovskite heterostructures, deduced from these UPS results, is presented in Figure 3c. The control 2D/3D perovskite exhibits a n‐doping surface after forming 2D perovskites, which is benefit to electron transfer. However, the high trap density at the surface of the control 3D perovskite likely accelerates charge recombination at the interface. Conversely, the planarization of the 3D perovskite results in a shift of the valence band maximum (VBM) and conduction band minimum (CBM) to shallower energy levels, attributed to the obvious changes in surface components. This energy band upshift in the planarized 3D perovskite facilitates the formation of a type‐II band alignment with the 2D perovskite, enabling electron transfer to the 2D layer while blocking holes with the energy barrier.

To investigate carrier dynamics within the 2D/3D perovskite heterostructure, we conducted steady‐state PL and time‐resolved photoluminescence (TRPL) measurements on glass substrates. As illustrated in Figure 3d, the planarized 3D perovskite exhibited a higher PL intensity compared to the control 3D perovskite sample, indicating that the surface planarization process effectively reduces defects in perovskites. Notably, the formation of a 2D capping layer further enhanced the PL intensity, reaching its highest value in the planarized 2D/3D perovskite heterojunction. The TRPL spectra in Figure 3e show a significant increase in charge‐carrier recombination lifetime from 9.72 ns in the control 3D PSCs, 44.65 ns in the planarized 3D PSCs, 158.74 ns in the control 2D/3D PSCs, and 301.91 ns in the planarized 2D/3D PSCs. These results indicate that both the surface planarization process and the 2D capping layer effectively reduce the surface defects in the perovskite, thereby suppressing nonradiative recombination.

The stability of the control and planarized 2D/3D perovskite films was analyzed by X‐ray diffraction (XRD) patterns, as illustrated in Figure 3f. Initially, the XRD patterns of the control 2D/3D perovskite films did not show any 2D perovskite component, and only displayed a small amount of PbI_2_ and perovskite diffraction peaks at 12.7° and 13.9°, respectively. However, after undergoing thermal aging at 100 °C for 120 min, the PbI_2_ diffraction peak in the control 2D/3D films became more pronounced, while the perovskite peak diminished. This suggests that the organic amine components (MA and FA) sublimate and escape from the perovskite thin films under thermal aging conditions. In contrast, the initial XRD pattern of the planarized 2D/3D perovskite displayed a characteristic diffraction peak at 5.2°, attributed to the n = 1 2D perovskite,^[^ ^17^ ^]^ in agreement with the GIWAXS results. Notably, the XRD spectrum of the aged planarized 2D/3D perovskite remained relatively unchanged. This indicates that the compact and uniform 2D capping layer effectively prevents the diffusion of organic amines from the 3D perovskites, thereby enhancing the stability of the planarized 2D/3D heterostructure.

The impact of a surface planarization‐epitaxial growth strategy on the photovoltaic performance was explored in PSCs based on a device structure of ITO/Me‐4PACz/Al_2_O_3_/1.57 eV 3D‐perovskite/2D‐perovskite/PCBM/C_60_/SnO_2_/Ag. We first investigated the effects of various types of planarization agents (including DMSO, dimethylformamide (DMF), and N‐Methylpyrrolidone (NMP)), the concentrations, and the PbI_2_ layer thickness on device performance. Figures S19 and S20 (Supporting Information) demonstrate that device performance is minimally influenced by the type and concentration of the planarization agent but primarily depends on the deposited PbI_2_ layer thickness. Consequently, a 0.5% DMSO in IPA (volume ratio) was selected as the planarization agent, and a 2D layer was formed using a 4 nm PbI_2_ layer.

**Figure 4:**
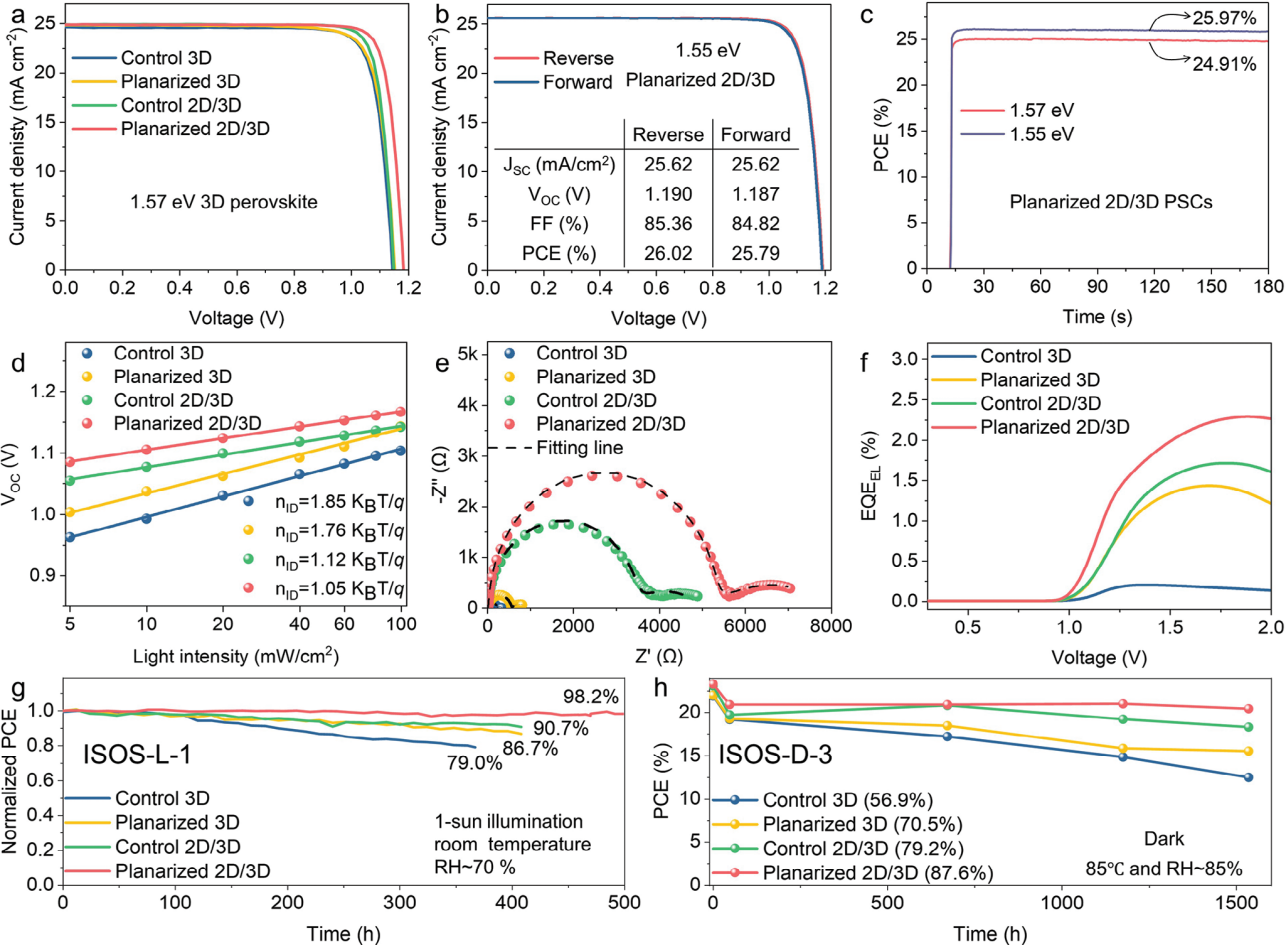
Performance of the PSCs based on the perovskites with a uniform 2D/3D heterojunction by the planarization‐epitaxial growth strategy. a) *J–V* curves of the champion control 3D, planarized 3D, control 2D/3D, and planarized 2D/3D p‐i‐n PSCs with a bandgap of 1.57 eV. b) *J–V* curves of the champion planarized 2D/3D p‐i‐n PSCs with a bandgap of 1.55 eV. c) The stabilized power outputs of the champion planarized 2D/3D PSCs. d) V_OC_ versus light intensity curves of the control 3D, planarized 3D, control 2D/3D, and planarized 2D/3D p‐i‐n PSCs. e) Nyquist plots of the control 3D, planarized 3D, control 2D/3D and planarized 2D/3D p‐i‐n PSCs. f) EQE_EL_ versus voltage of the control 3D, planarized 3D, control 2D/3D, and planarized 2D/3D p‐i‐n PSCs. g) Stability of encapsulated PSCs under ISOS‐L‐1 protocol (1‐sun illumination using LEDs source, ambient condition with RH of 70%). The initial PCEs of the devices based on control 3D, planarized 3D, control 2D/3D and planarized 2D/3D are 22.07%, 22.89%, 23.52%, and 24.57%, respectively. h) Stability of encapsulated PSCs under ISOS‐D‐3 protocol.

**Figure** **4**a shows the typical current density–voltage (J–V) curves of PSCs with an area of 0.1 cm^2^. The J‐V curves show that the polished 2D/3D PSCs delivered a champion PCE of 25.31%, with an open circuit voltage (V_OC_) of 1.18 V, a short‐circuit current density (J_SC_) of 24.86 mA cm^−2^, and a fill factor (FF) of 86.0%. The external quantum efficiency (EQE) spectra and corresponding 1st derivative spectrum are displayed in Figure S21 (Supporting Information). The integrated J_SC_ values are 23.45 mA cm^−2^ for the 2D/3D PSCs, and the bandgap of the 3D perovskite component within this device is further confirmed to be 1.57 eV. Compared with the control 3D, planarized 3D, and control 2D/3D PSCs, the efficiency improvement of planarized 2D/3D PSCs mainly results from the enhanced V_OC_ and FF (Figure S22, Supporting Information), which is attributed to the synergistic passivation effect of the surface planarization and 2D perovskite. More impressively, the planarized 2D/3D PSCs achieve a record fill factor of 87.6%, as shown in Figure S23 (Supporting Information). The champion planarized 2D/3D PSCs also show negligible J–V hysteresis (Figure S24, Supporting Information) and a stabilized power output of 24.91% under a bias voltage of 1.057 V for 160 s (Figure 4c). Furthermore, the PCE of planarized 2D/3D PSCs can be further improved to 26.02% (Figure 4b) by employing 1.55 eV 3D‐perovskite (Figure S25, Supporting Information) and maintains a stabilized power output of 25.97% under a bias voltage of 1.056 V for 160 s (Figure 4c). The detailed photovoltaic parameters of 1.57 and 1.55 eV PSCs are summarized in Table S3 (Supporting Information).

To further elucidate the enhanced V_OC_ and FF attributed to the planarization‐epitaxial growth strategy, we analyzed the ideality factor of PSCs by examining their voltage dependence under varying light intensities. Figure 4d demonstrates that the slope of V_OC_ versus light intensity is calculated to be 1.85, 1.76, 1.12, and 1.05 for the control 3D, planarized 3D, control 2D/3D, and planarized 2D/3D PSCs, respectively. This trend indicates decreased trap‐induced recombination under open‐circuit conditions for the planarized devices. Additionally, the charge‐recombination dynamics of these devices were explored through electrochemical impedance spectroscopy (EIS), conducted under an external voltage of 1.0 V. As depicted in Figure 4e and Figure S26 (Supporting Information), the planarized 2D/3D PSCs exhibit the highest recombination resistance. This elevated recombination resistance plays a pivotal role in minimizing nonradiative recombination losses, a fact corroborated by the electroluminescence (EL) efficiency (EQE_EL_) presented in Figure 4f. The planarized 2D/3D PSCs display the highest EQE_EL_, indicating minimal nonradiative recombination losses. These remarkably low nonradiative recombination losses in the planarized 2D/3D PSCs are a primary factor contributing to their high V_OC_ and FF. Generally, such nonradiative recombination losses are attributed to defects at the 2D and 3D interfaces. The reduced nonradiative recombination losses in the planarized 2D/3D PSCs suggest that surface planarization effectively mitigates these interfacial defects, enhancing device performance.

In addition to the device performance, assessing the operational stability of PSCs is crucial for their practical deployment. We tested the long‐term operational stability of our devices following the ISOS‐L‐1 and ISOS‐D‐3 protocols.^[^ ^44^ ^]^ The devices were encapsulated with a glass using epoxy, as shown in Figure S27a (Supporting Information). Under the ISOS‐L‐1 protocol (Figure 4g), the planarized 2D/3D PSCs demonstrated remarkable stability, retaining 98.2% of the initial PCE after 500 h of continuous illumination. This performance surpasses that of control 3D, planarized 3D, and control 2D/3D PSCs. Following the ISOS‐D‐3 protocol (Figure 4h), the PSCs maintained their initial PCE at 56.9%, 70.5%, 79.2%, and 87.6% after 1536 h for control 3D, planarized 3D, control 2D/3D, and planarized 2D/3D, respectively. This suggests that the 2D/3D bilayer perovskite gains inherent stability by adding a homogeneous and uniform 2D capping layer. The enhanced stability of the planarized 2D/3D PSCs under the ISOS‐D‐3 protocol is further evidenced by their optical images (Figure S27b, Supporting Information). After 600 h of aging, the color of region ① (without covering glass) in control 3D, planarized 3D, and control 2D/3D PSCs turned yellow, whereas the same region in the planarized 2D/3D PSCs remained black. This indicates that a homogeneous and compact 2D capping layer significantly improves device stability, underscoring the effectiveness of the surface planarization‐epitaxial growth strategy in fabricating stable PSCs.

**Figure 5:**
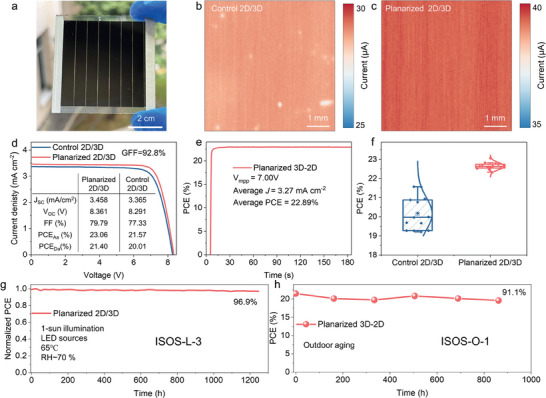
Homogeneous charge extraction in planarized 2D/3D PSMs. a) Optical photo of the planarized 2D/3D PSM. b,c) The photocurrent images of (b) control 2D/3D and (c) planarized 2D/3D PSM. d) *J–V* curves of the champion control 2D/3D and planarized 2D/3D PSMs with seven subcells connected in series. e) The stabilized power output of the champion planarized 2D/3D PSM. f) PCE_Aa_ distribution of the control 2D/3D and planarized 2D/3D PSMs. g) Stability of encapsulated planarized 2D/3D PSM under ISOS‐L‐3 protocol (1‐sun illumination using LEDs source at 65 °C with RH of 70%). The initial PCE_Aa_ of planarized 2D/3D PSM is 22.53%. (h) Stability of encapsulated planarized 2D/3D PSM under ISOS‐O‐1 protocol (The solar module is stored outdoors, and its PCE_Aa_ was acquired using a solar simulator every two weeks).

To evaluate the scalability of our surface planarization‐epitaxial growth strategy for forming a homogeneous and compact 2D capping layer via a vapor‐assisted two‐step process, we fabricated PSMs consisting of seven sub‐cells arranged in series on 6 cm × 6 cm ITO substrates. The optical image of the module is depicted in **Figure** **5**a. The laser patterning of P1, P2, and P3 lines is shown in Figure S28 (Supporting Information). The PSM was designed with a subcell width of 6.8 mm and a dead‐area width of 0.49 mm, resulting in a geometric fill factor of 92.8%. The above characterization results consistently show that planarized 3D perovskites are conducive to forming a homogeneous and compact 2D capping layer on their surface. Meanwhile, the 2D capping layer formed on the surface of control 3D perovskites tends to be discontinuous. The uniformity of this 2D capping layer significantly influences the homogeneity of carrier extraction within the device, which can be directly observed by the photocurrent images. Figure 5b,c illustrate a stark contrast in the photocurrent mapping of control 2D/3D devices compared to their planarized counterparts. In the control 2D/3D device, there is a clear spatial inhomogeneity visible in the photocurrent map, characterized by distinct bright and dark regions that indicate localized areas of lower and higher photocurrent, respectively. These areas appear to be unevenly distributed, suggesting variations in carrier extraction efficiency across the device. In contrast, the photocurrent of the planarized 2D/3D device presents a more uniform and higher current compared to the control. It exhibits only moderate fluctuations in photocurrent, indicating a more homogeneous carrier extraction process across the entire device.

Figure 5d displays the *J–V* curves for 2D/3D PSMs under AM 1.5G 100 mW cm⁻^2^ illumination. As expected from the photocurrent mapping analysis, the planarized 2D/3D PSM demonstrates an enhanced active area power conversion efficiency (PCE_Aa_) of 23.06% with an active area of 22.8 cm^2^, corresponding to a designated area PCE (PCE_Da_) of 21.4%. A PSM with a PCE_Da_ of 20.78% was sent to the National PV Industry Measurement and Testing Center for efficiency certification and the certified PCE_Da_ reached 20.75% for the PSM with a designated area of 24.04 cm^2^, as shown in Figure S29 and S30 (Supporting Information). The stabilized maximum power point tracking of the module efficiency was certified 20.18% (Figure S31, Supporting Information). The *J–V* curves of this planarized 2D/3D PSM, as explored under different dwell times and scan directions, exhibit negligible hysteresis (Figure S32, Supporting Information). Stabilized power output measurements further confirm the module's high performance, with a stabilized PCE_Aa_ of 22.89% as illustrated in Figure 5e. Owing to the uniform carrier extraction in the planarized 2D/3D perovskite, the solar module achieved a high FF of 81%, as shown in Figure S33 (Supporting Information). Figure 5f provides a comparative statistical analysis of PCEs from control and planarized 2D/3D PSMs. Notably, the surface planarization has led to an improvement in device reproducibility. This improvement is demonstrated by the narrower distribution of PCE values, as indicated by the reduced statistical deviations.

The stability of the planarized 2D/3D PSM was evaluated following the ISOS‐L‐3 and ISOS‐O‐1 protocols. The 6 cm × 6 cm PSM was encapsulated by a 7 cm × 7 cm glass using epoxy. For assessing long‐term operational stability under ISOS‐L‐3 conditions, the planarized 2D/3D PSM demonstrated a commendable performance retention. After 1246 h of continuous illumination at 65 °C, the module's efficiency slightly decayed to 96.9% of its original value. The result in Figure 5g signifies excellent stability under elevated temperature conditions. In terms of outdoor stability, tested under the ISOS‐O‐1 protocol, the encapsulated planarized 2D/3D PSM was exposed to real‐world outdoor conditions without additional protective measures. Periodic performance evaluations were conducted in the laboratory using a solar simulator. As illustrated in Figure 5h, the module maintains 91.1% of its initial efficiency after 862 h of outdoor exposure.

## 3 Conclusion

In conclusion, our study successfully demonstrates the fabrication of a uniform 2D/3D perovskite heterojunction, where interfacial defects are significantly mitigated through a surface planarization strategy. This approach has led to the construction of a highly stable, uniform 2D/3D perovskite heterostructure, achieving an efficiency of 26.02% in PSCs. Additionally, for planarized 2D/3D PSMs with an active area of 22.8 cm^2^, we have attained an impressive efficiency of 23.06%. The longevity and durability of this PSM are further underscored by its performance under prolonged operational conditions. After 1246 h of continuous operation following the ISOS‐L‐3 protocol, the device remarkably retains 96.9% of its initial efficiency. The surface planarization strategy demonstrates the positive outcomes of generating flatter and uniform heterojunctions toward efficient and stable large‐area perovskite‐based photovoltaic technology, prompting us to foresee further related research in this field.

## Conflict of Interest

The authors declare no conflict of interest.

## Supporting information



Supporting Information

Supporting Information

## Data Availability

The data that support the findings of this study are available from the corresponding author upon reasonable request.
