# Supplementary material for: Surface Planarization‐Epitaxial Growth Enables Uniform 2D/3D Heterojunctions for Efficient and Stable Perovskite Solar Modules
Source: Adv Sci (Weinh). 2024 Nov 3;12(1):2407380. doi: 10.1002/advs.202407380 (PMC11714145; doi:10.1002/advs.202407380)
Supplement: Supplementary file 1 — Supporting Information [file ADVS-12-2407380-s001.pdf]

ADVANCED SCIENCE
Open Access

Supporting Information

for *Adv. Sci.*, DOI 10.1002/advs.202407380

Surface Planarization-Epitaxial Growth Enables Uniform 2D/3D Heterojunctions for Efficient and Stable Perovskite Solar Modules

Dongxu Lin, Jun Fang, Sibo Li, Zhenye Zhan, Huan Li, Xin Wang, Guanshui Xie, Daozeng Wang, Nuanshan Huang, Haichen Peng, Weiguang Xie, Luis K. Ono, Yabing Qi and Longbin Qiu**

Surface Planarization-Epitaxial Growth Enables Uniform 2D/3D Heterojunctions for Efficient and Stable Perovskite Solar Modules

Dongxu Lin[1†], Jun Fang[1†], Sibo Li[1], Zhenye Zhan[2], Huan Li[1], Xin Wang[1], Guanshui Xie[1], Daozeng Wang[1], Nuanshan Huang[1], Haichen Peng[1], Weiguang Xie[2], Luis K. Ono[3], Yabing Qi[4,], Longbin Qiu[1,*]*

[1] *Shenzhen Key Laboratory of Intelligent Robotics and Flexible Manufacturing Systems, Department of Mechanical and Energy Engineering, SUSTech Energy Institute for Carbon Neutrality, Southern University of Science and Technology, Shenzhen, 518055, China*

[2] *College of Physics & Optoelectronic Engineering, Jinan University, Guangzhou, 510632, Guangdong, China*

[3] *Energy Materials and Surface Sciences Unit (EMSSU), Okinawa Institute of Science and Technology Graduate University (OIST), 1919-1 Tancha, Onna-son, Kunigami-gun, Okinawa 904-0495, Japan*

[4] *Global Institute of Future Technology, Shanghai Jiao Tong University, Shanghai 200240, P. R. China*

*Corresponding authors: Yabing Qi, Email: yabingqi@sjtu.edu.cn; Longbin Qiu, Email: qiulb@sustech.edu.cn

[†] These authors contributed equally to this work.

Experimental section

Materials

Dimethyl sulfoxide (DMSO) and dimethylformamide (DMF) were purchased from Alfa Aesar. Isopropanol (IPA), ethyl alcohol, aluminum oxide nanoparticles (< 50 nm particle size, 20 wt% in IPA), and chlorobenzene (CB) were purchased from Sigma Aldrich. Lead iodine (PbI_2, 99.99%), cesium iodide (CsI), and phenyl-C61-butyric acid methyl-ester ($PC_{61}BM$) were purchased from Advanced Election Technology. Formamidine iodide (FAI), methylammonium bromide (MABr), and methylammonium iodide (MAI) were purchased from Greatcell Solar Materials. (4-(3,6-Dimethyl-9H-carbazol-9-yl)butyl)phosphonic acid (Me-4PACz) was purchased from TCI. Phenethylammonium bromide (PEABr), fullerene (C_{60}), and bathocuproine (BCP) were purchased from Xi'an Polymer Light Technology. All chemicals were utilized in their as-received state without undergoing additional purification.

Materials Preparations

Preparation of planarization agent: The planarization agent was prepared by mixing DMSO and IPA with a volume ratio of 1:200. The planarization agent was put into a Vortex Shaker for 30 min before use.

Preparation of perovskite solutions: For the 1.57 eV band gap 3D perovskite, 1.52 M $Cs_{0.05}MA_{0.1}FA_{0.85}PbI_{2.9}Br_{0.1}$ was dissolved in 1 mL mixed solvent of DMF and DMSO with a volume ratio of 3:1. For the 1.55 eV band gap 3D perovskite, 1.62 M $Cs_{0.05}MA_{0.1}FA_{0.85}PbI_3$ was dissolved in 1 mL mixed solvent of DMF and DMSO with a volume ratio of 3:1. The perovskites precursor solutions were vibrated overnight by using a Vortex Shaker. Note that the precursor solutions were filtered using a 0.22 μm filter before deposition.

Device fabrication

For the small-area perovskite solar cells, the perovskite films were fabricated using a one-step antisolvent method in an N_2-filled glovebox. The glass/ITO substrates were treated using UV-ozone for 30 min and then quickly transferred to the N_2-filled glovebox. Then, 70 μL of Me-4PACz (0.4 mg mL^{-1} in ethyl alcohol) was spin-coated at 3000 rpm for 30 s and annealed at 100 °C for 10 min. Next, 100 μL diluted Al_2O_3 dispersion solution (the Al_2O_3 dispersion solution was prepared by diluting 15 μL Al_2O_3 solution in 2.25 mL IPA.) was spin-coated on the Me-4PACz film at 5000 rpm for 30 s and heated at 100 °C for 10 min. For deposition of perovskite films, the perovskite precursor solution was spin-coated on the ITO/Me-4PACz/Al_2O_3 substrates at 5000 rpm for 45 s and 200 μL CB was dropped in the center of spinning substrates at 20 s after starting the program. The precursor films were annealed at 65 °C for 5 min and 105 °C for 20 min to obtain the control 3D perovskite film. For planarized 3D perovskite film, 50 μL planarization agent was spun on the above 3D perovskite at 5000 rpm for 30 s and heated at 100 °C for 5 min. For the control and planarized 2D/3D perovskite heterostructures, a 4 nm-thick layer of PbI_2 was evaporated at a rate of 0.2 Å s^{-1} onto the control and planarized 3D perovskites in a vacuum chamber and then 100 μL PEABr solution was spun on the above control 3D/PbI_2 and planarized 3D/PbI_2 film at 5000 rpm for 30 s and annealed at 100 °C for 5 min. After cooling down to room temperature, the PCBM solution with a concentration of 10 mg mL^{-1} in CB was deposited by 3000 rpm for 30 s. Finally, the 10 nm C_{60} (thermal evaporation), 10 nm SnO_x (Atomic Layer Deposition, ALD) and 90 nm of metal electrode Ag (thermal evaporation) were sequentially deposited on the surface of PCBM to obtain the complete devices. The mask for determining the photoactive area of the PSCs was 0.1 cm^2.

For the perovskite solar modules, the P1, P2, and P3 lines were obtained by laser scribing (532 nm). The fabrication is analogous to small-area solar cells except for the amount of solution used in the preparation process. More dosage of the solution is needed to completely cover the 6 cm × 6 cm substrate.

Characterization

Grazing incidence X-ray diffraction (GIXRD) patterns were acquired in air by using a Rigaku Smartlab with Cu Kα radiation in the 2θ range of 3-40° at a scanning rate of 10° min^{-1}. UPS measurements were carried out on PHI 5000 Versaprobe III using monochromatized He I radiation at 21.22 eV. The photoluminescence (PL) spectra and time-resolved PL (TRPL) were performed using an Edinburgh Instrument FLS1000 system applying a 450 nm laser as the excitation source. The scanning electronic microscope (SEM) images were obtained from Apreo2 S Lovac field emission SEM at an acceleration voltage of 3 kV. The contact angle measurements of the films were conducted on a KRUSS-The Drop Shape Analyzer DSA25 system and water was used as the wetting solvent. The roughness of the films was collected from the atomic force microscope (AFM) (Bruker, Dimension Edge). The AFM-IR images were conducted on NanoIR3s, Bruker. The absorption of the films was attained from UV-vis spectroscopy (HITACHI, UH5700), employing an optical range from 300 to 900 nm in air. The Electrochemical impedance spectroscopy (EIS) curves of the devices were determined by using an Autolab electrochemical station with a frequency of 0.1 Hz to 1 MHz in a dark environment with an external voltage of 1.0 V. The electroluminescence (EL) spectra were characterized by a Keithley 2420 source meter and integrating sphere connected to a spectrophotometer (QE65Pro).

The current density versus voltage (J-V) characteristics of the devices were tested using a Keithley 2420 source meter under AM 1.5G one-sun illumination (100 $mW\cdot cm^{-2}$) which was produced by a solar simulator (Sol3A Class AAA, Oriel, Newport, USA) in air. A standard reference silicon cell (91150-KG3, Newport, USA) was used to calibrate the light intensity. The metal mask with an area of 0.1 cm^2 was employed to determine the active area of the PSCs. Both the reverse and forward scan of J-V curves were tested with 30 ms dwell time. The incident photon to converted electron efficiency (IPCE) measurement was conducted to obtain the external quantum efficiency (EQE) spectra with a range from 300 to 850 nm using the EQE system (IQE 200B, Newport).

For the operation stability measurement of ISOS-L-1 protocol, encapsulated PSCs were carried out at the MPP using a white light LED with the intensity calibrated to satisfy one-sun conditions. The PSCs were tested in a chamber at ambient atmosphere and temperature. For the operation stability measurement of ISOS-D-3 protocol, encapsulated PSCs were aged at 85 °C and RH~85% chamber in a dark environment. For the operation stability measurement of ISOS-L-3 protocol, encapsulated planarized 2D/3D perovskite solar module was tested on a heater with temperature of ~65 °C at the MPP condition under a white light LED source. For the operation stability measurement of ISOS-O-1 protocol, encapsulated planarized 2D/3D perovskite solar module was exposed to real-world outdoor conditions without additional protective measures, and its PCE was characterized using a solar simulator every two weeks.

Supplementary Note S1

The surface energy of the perovskite film was computed employing the Owens-Wendt method, taking into account the polar and dispersive components of surface energy. The combination of these components results in the total surface energy of the solid perovskite film represented as σ_S.

$$\sigma_S = \sigma_S^D + \sigma_S^P$$

Where σ_S^D and σ_S^P represent the dispersive and polar components, respectively, of the surface energy of the solid perovskite film.

For Owens-Wendt primary equation

$$\sqrt{\sigma_l^D + \sigma_S^D} + \sqrt{\sigma_l^P + \sigma_S^P} = \frac{\sigma_l(1 + \cos\theta)}{2}$$

where σ_l^D and σ_l^P are the dispersive and polar components of the surface tension of the liquid respectively. σ_l represents the total surface tension of the liquid. The first step in calculating surface energy involves measuring the contact angle of a purely dispersive liquid. Diiodomethane is commonly used for this purpose, as it possesses virtually no polar component in its surface tension, primarily due to molecular symmetry. This results in $\sigma_l = \sigma_l^D$= 50.8 mN/m. Consequently, the Owens-Wendt primary equation simplifies to:

$$\sigma_S^D = \frac{\sigma_l(1 + \cos\theta)^2}{4}$$

By applying this equation, the dispersive component of the surface energy of the solid perovskite film, σ_S^D, can be directly determined once the contact angle of diiodomethane on the perovskite film is measured. The subsequent step involves measuring the contact angle using a liquid with known dispersive and polar components. Glycerol is typically used for this purpose, with its polar component σ_l^P= 26.4 mN/m and its dispersive component σ_l^D = 37.0 mN/m. Inserting these values into the Owens-Wendt primary equation, alongside the liquid's surface tension and the previously calculated σ_S^D, enables the calculation of the polar component of the solid's surface energy, σ_S^P. The total surface energy of the solid perovskite film, σ_S, is then the sum of σ_S^D and σ_S^P.

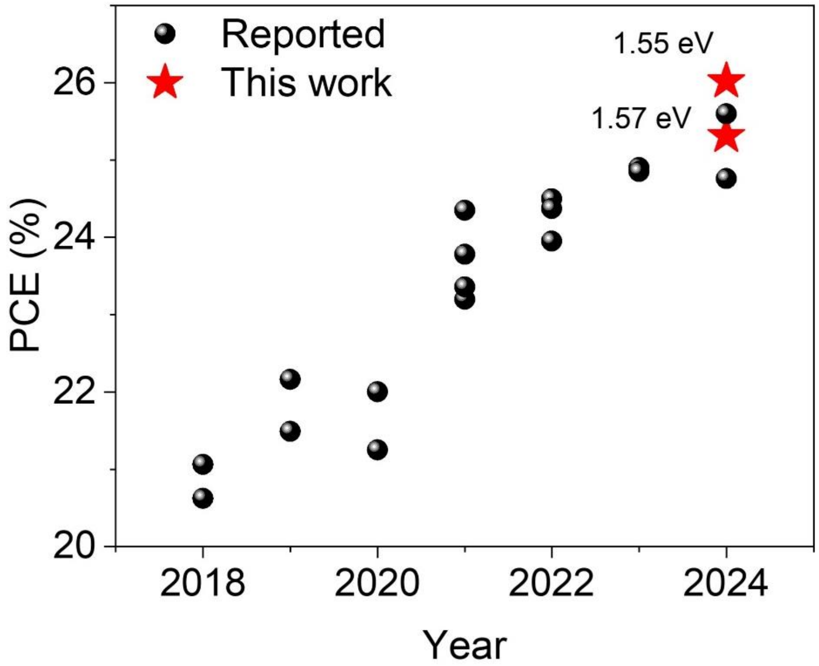

Figure S1. Comparison of PCEs of the 2D/3D PSCs from the literature and our results in this work.

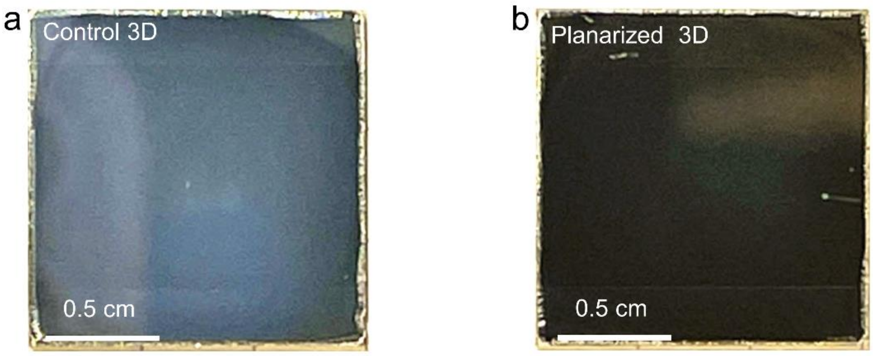

Figure S2. Optical images of (a) the control 3D perovskite (without planarization) and (b) planarized 3D perovskites.

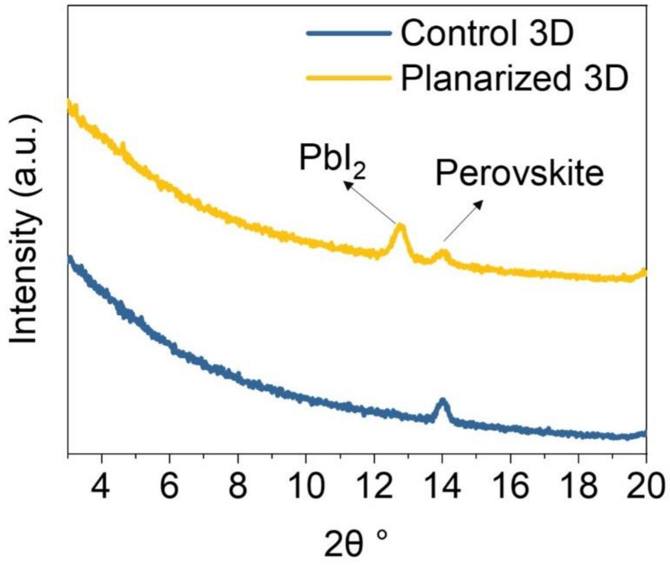

Figure S3. GIXRD patterns of the control and planarized 3D perovskites at the incidence angle of 0.1°.

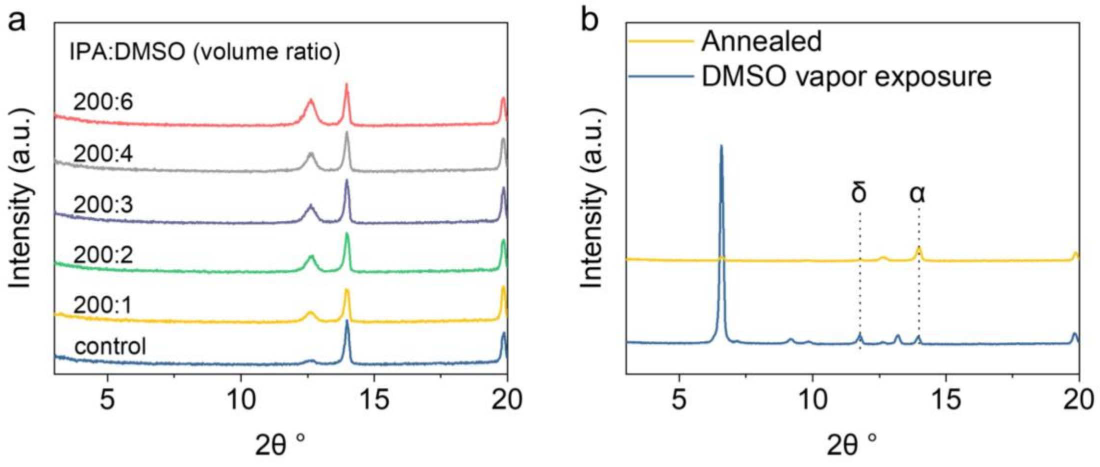

Figure S4. XRD patterns of (a) 3D perovskites treated with various DMSO concentrations in IPA and (b) 3D perovskite directly exposed to DMSO vapor followed by annealing.

As shown in **Figure S4a**, even when the DMSO concentration in the planarization agent was increased sixfold to 200:6 (volume ratio of IPA:DMSO), no XRD diffraction peaks corresponding to the δ-phase perovskite were observed, confirming that low DMSO concentrations do not induce a phase transition. To observe the effect of higher DMSO content, the perovskite film was directly exposed to 200 μL of DMSO vapor for 5 min, and the XRD results demonstrated the formation of intermediate and δ-phases, as shown in **Figure S4b**. However, most of these phases can be converted to the α-phase perovskite after annealing at 100 °C for 5 min.

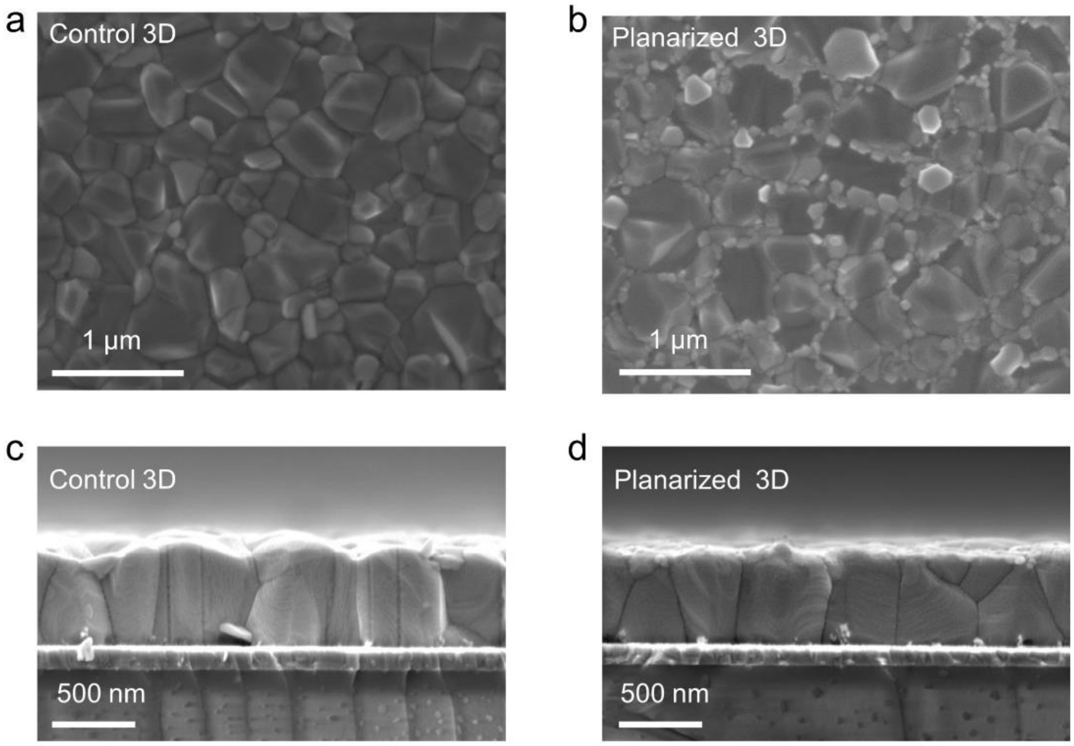

Figure S5. Top surface SEM images of (a) the control 3D perovskite and (b) planarized 3D perovskite. Cross-sectional SEM images of (c) the control 3D perovskite and (d) planarized 3D perovskite.

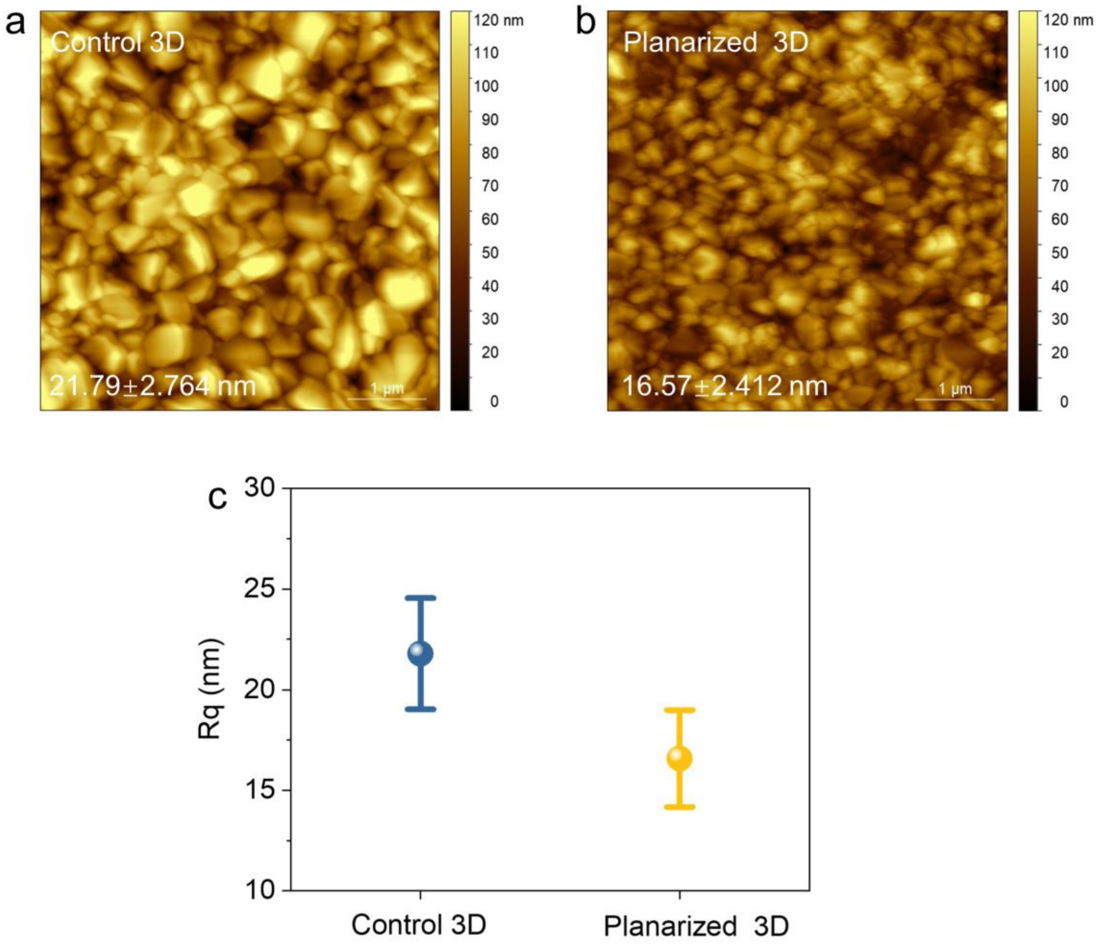

Figure S6. AFM images of (a) the control 3D and (b) planarized 3D perovskite films. (c) surface roughness of the control and planarized 3D perovskites.

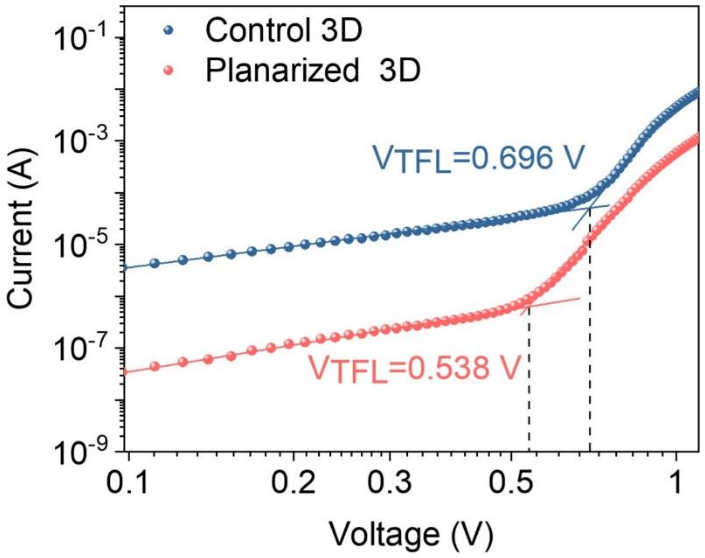

Figure S7. The SCLC curves of the hole-only devices based on the control and planarized 3D perovskites.

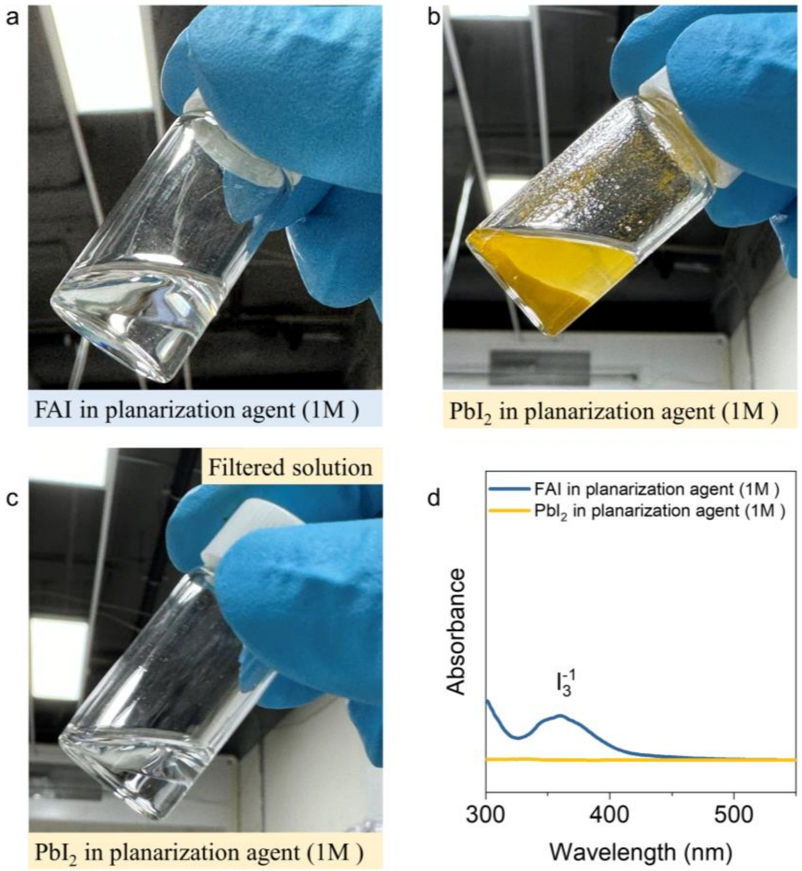

Figure S8. Optical photos of (a) FAI dissolved in the planarization agent, (b) PbI_2 dissolved in the planarization agent, and (c) the filtered solution of PbI_2 dissolved in the planarization agent. (d) The UV-Vis spectra of FAI and PbI_2 solutions.

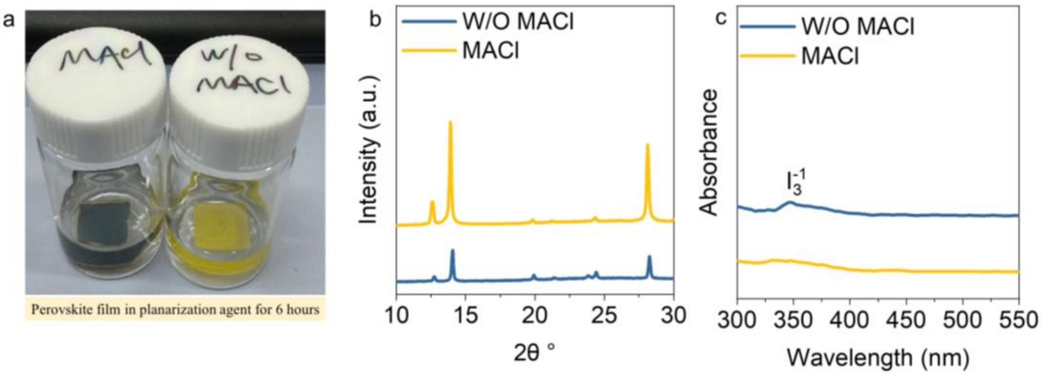

Figure S9. (a) Optical photo of perovskite film with and without MACl immersed in the planarization agent for 5 hours. (b) XRD patterns of perovskite film with and without MACl before immersion. (c) UV-Vis spectra of the planarization agent after perovskite film immersion.

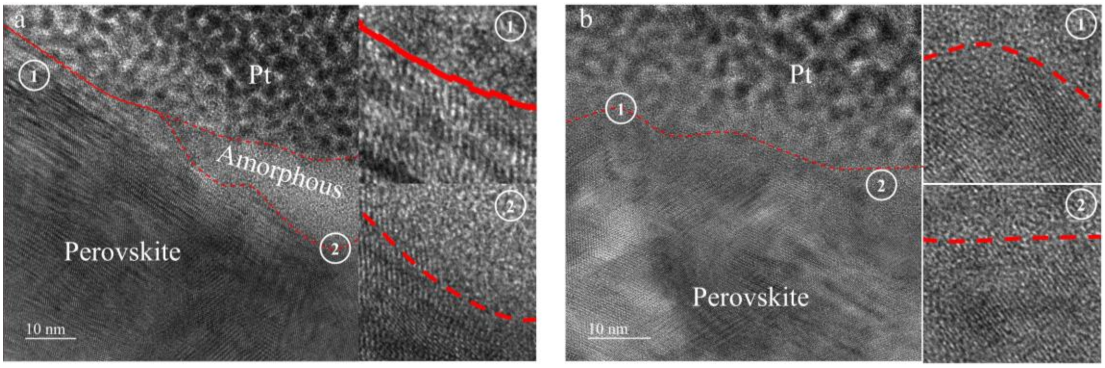

Figure S10. Cross-sectional HRTEM of (a) control 3D and (b) planarized 3D perovskites.

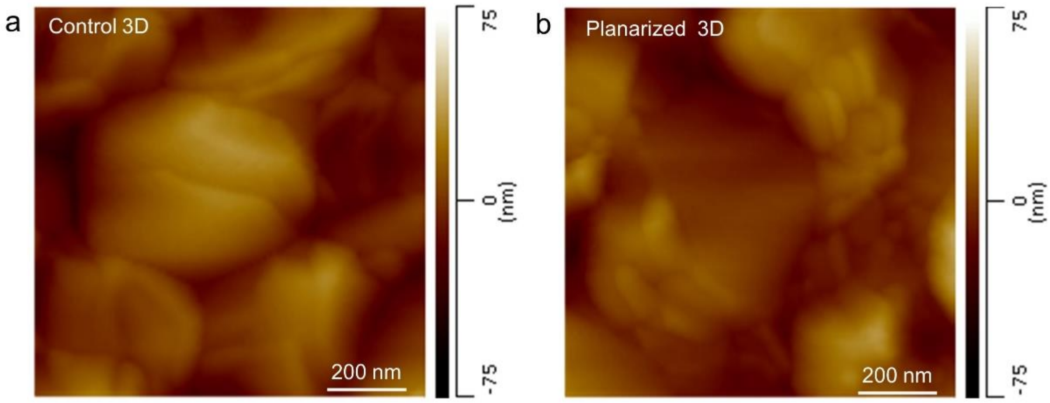

Figure S11. AFM images of (a) the control 3D and (b) planarized 3D perovskite films for collecting the infrared information.

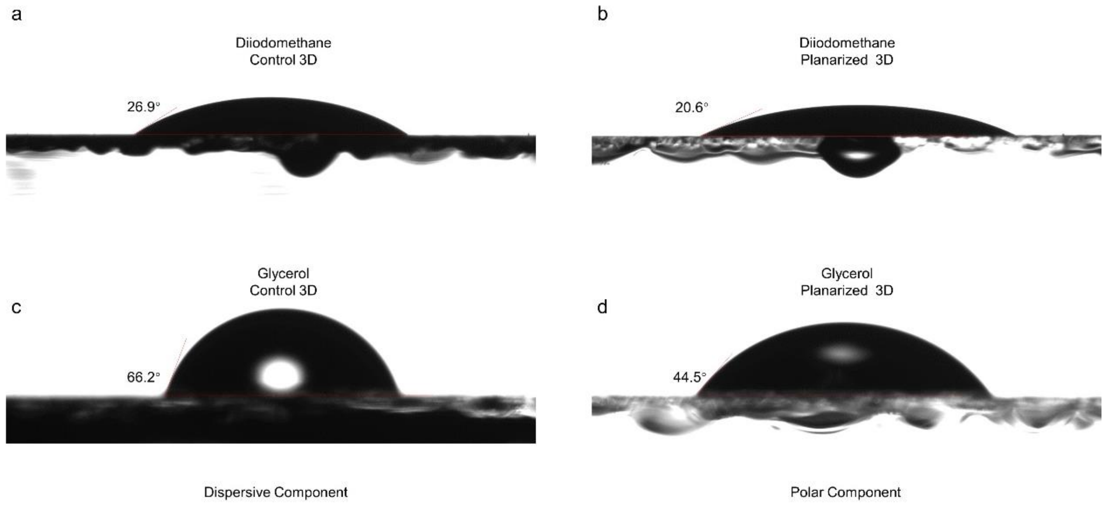

Figure S12. The contact angle of diiodomethane on (a) the control 3D and (b) planarized 3D perovskite films. The contact angle of glycerol on (c) the control 3D and (d) planarized 3D perovskite films.

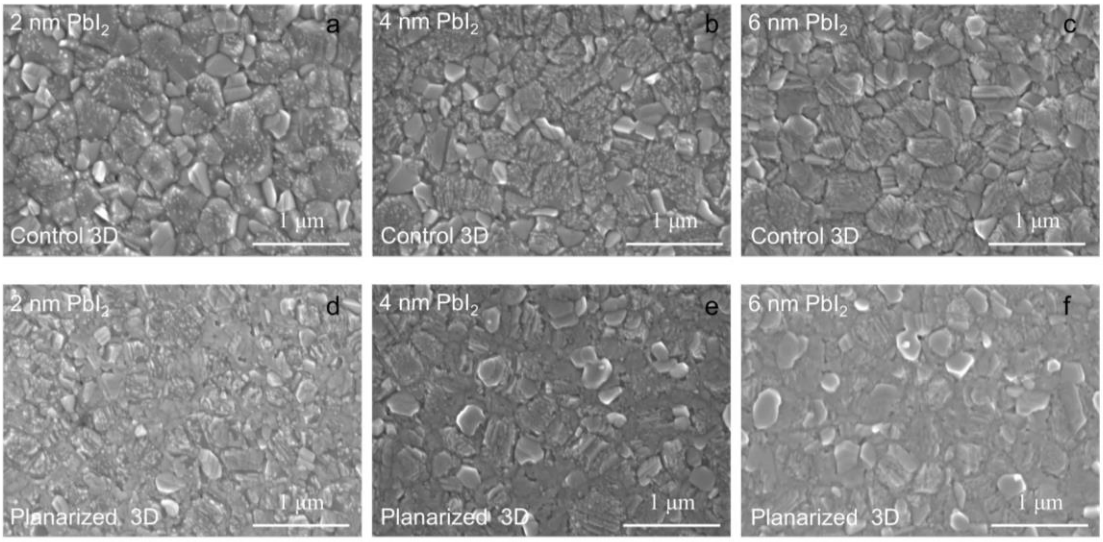

Figure S13. Top surface SEM images of the control 3D perovskites after vapor depositing (a) 2 nm, (b) 4 nm, and (c) 6 nm PbI_2, and planarized 3D perovskites after vapor depositing (d) 2 nm, (e) 4 nm, and (f) 6 nm PbI_2. Note: The average thickness of the vapor-deposited PbI_2 was determined using a calibrated quartz crystal microbalance (QCM).

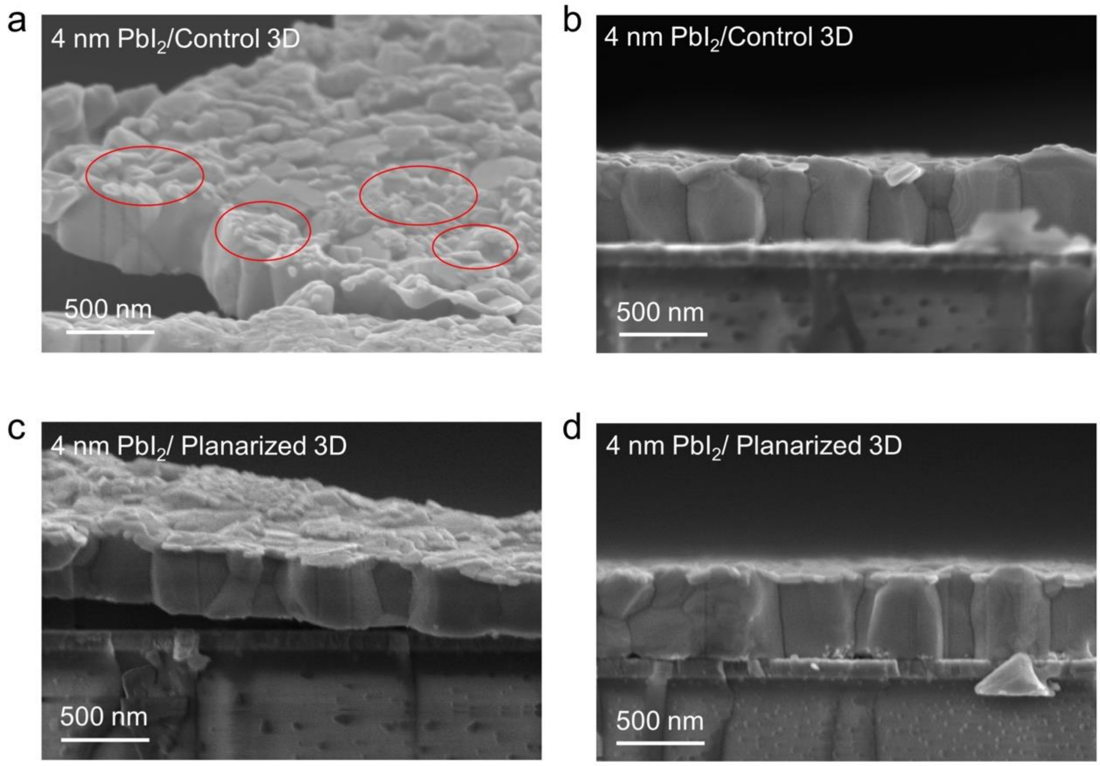

Figure S14. Cross-sectional SEM images of (a)-(b) the control 3D perovskites and (c)-(d) planarized 3D perovskites after vapor deposition of 4 nm PbI_2.

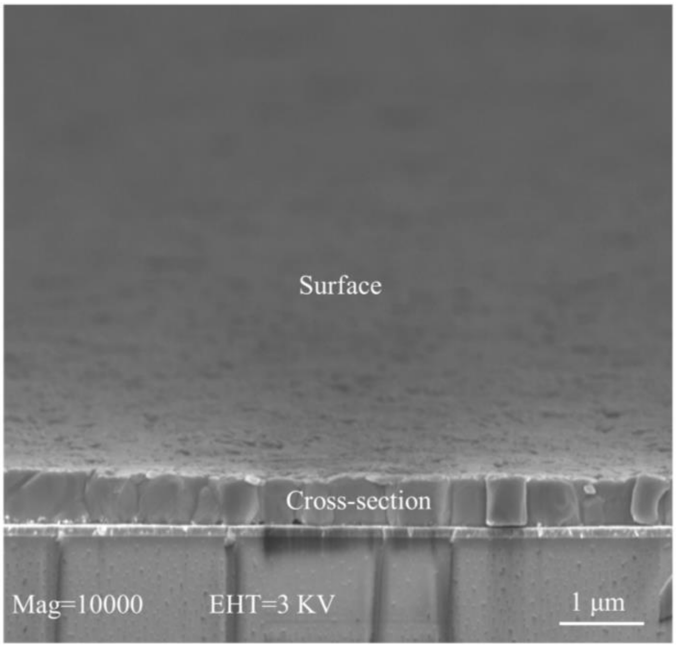

Figure S15. The cross-sectional SEM of planarized 3D perovskites after vapor deposition of 4 nm PbI_2 that captures both the surface and cross-sectional morphology simultaneously.

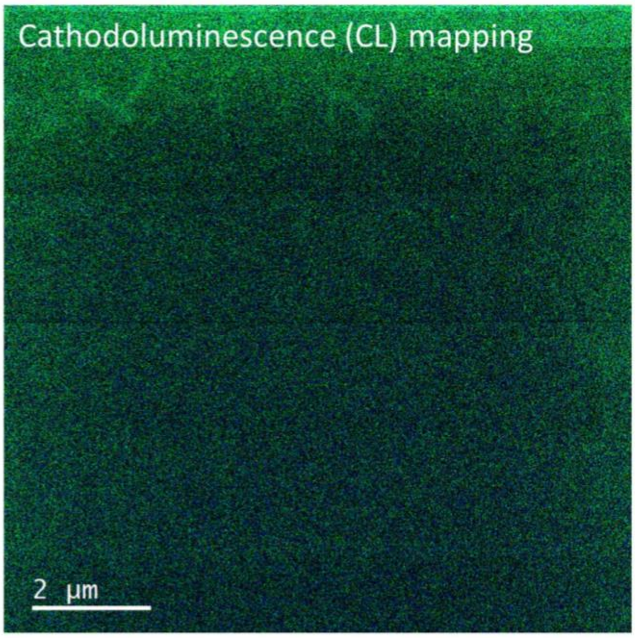

Figure S16. Cathodoluminescence (CL) mapping of planarized 3D perovskites after vapor deposition of 4 nm PbI_2. The spatial distribution of green emission was recorded from 530 to 590 nm.

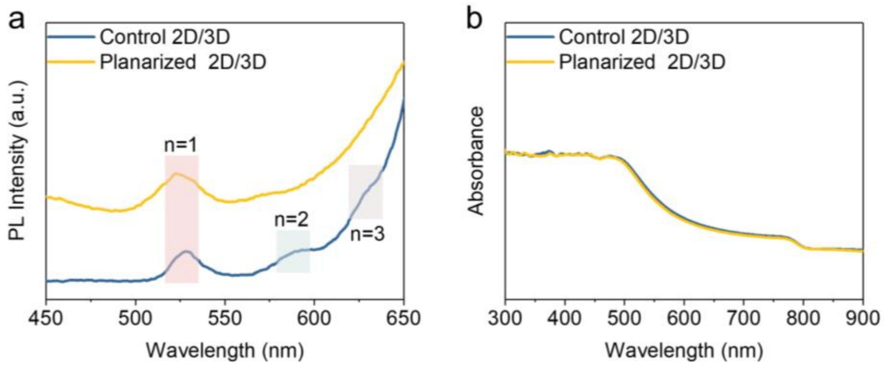

Figure S17. (a) PL spectra of control 2D/3D and planarized 2D/3D perovskites under 405 nm laser excitation for surface emission. (b) UV-Vis spectra of control 2D/3D and planarized 2D/3D perovskites.

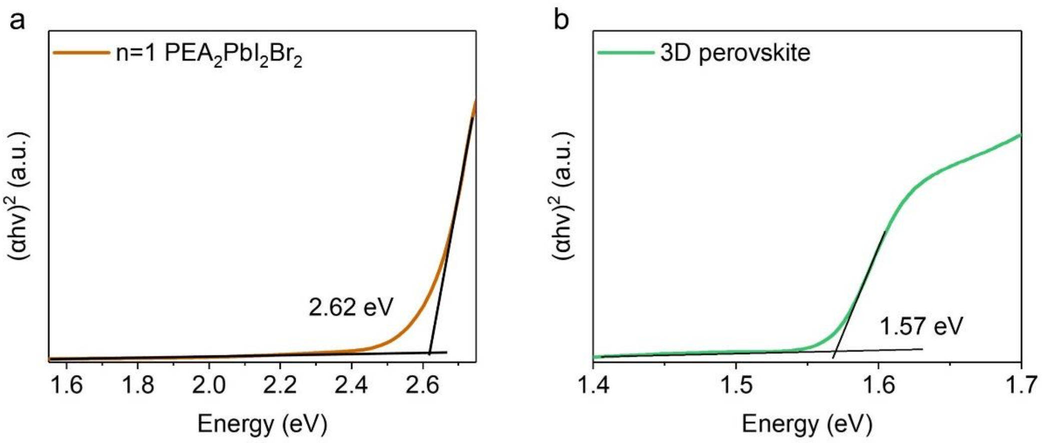

Figure S18. Tauc plot curves of (a) 2D n=1 $PEA_2PbI_2Br_2$ and (b) 3D $Cs_{0.05}MA_{0.1}FA_{0.85}PbI_{2.9}Br_{0.1}$ perovskites.

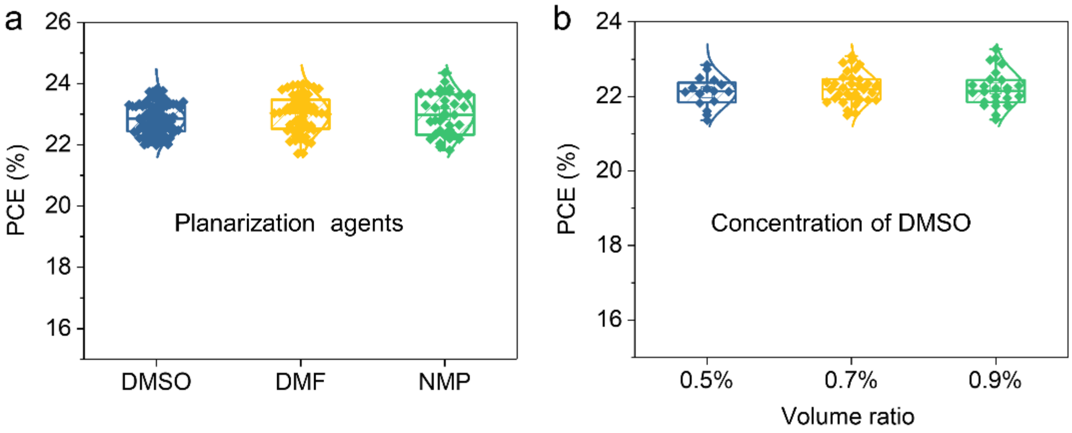

Figure S19. PCE distribution of the planarized 2D/3D PSCs using (a) different planarization agents and (b) different concentrations of DMSO in IPA.

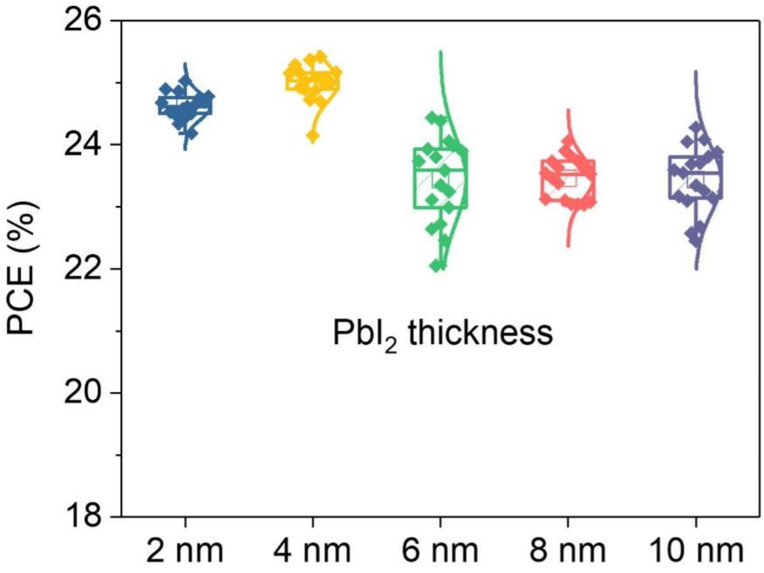

Figure S20. PCE distribution of the planarized 2D/3D PSCs with different PbI_2 thicknesses.

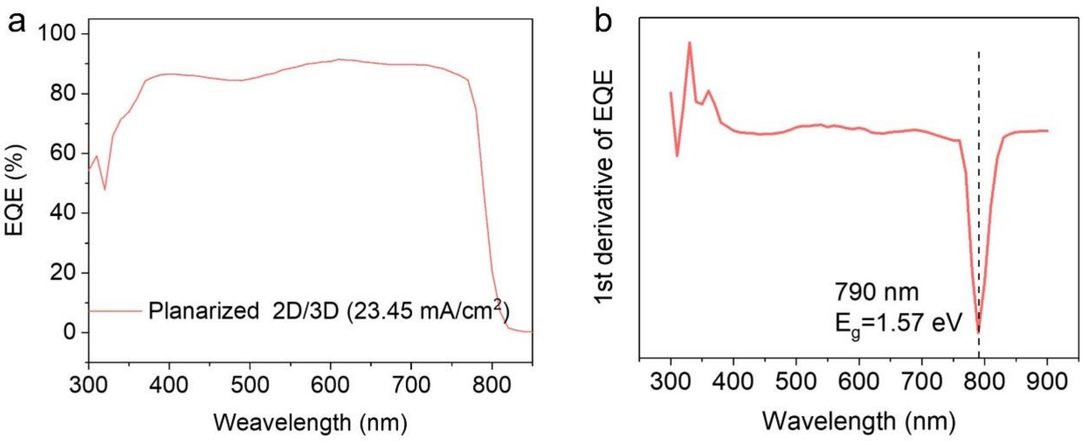

Figure S21. (a) EQE spectrum of the planarized 2D/3D PSC and (b) its corresponding 1st derivative spectrum.

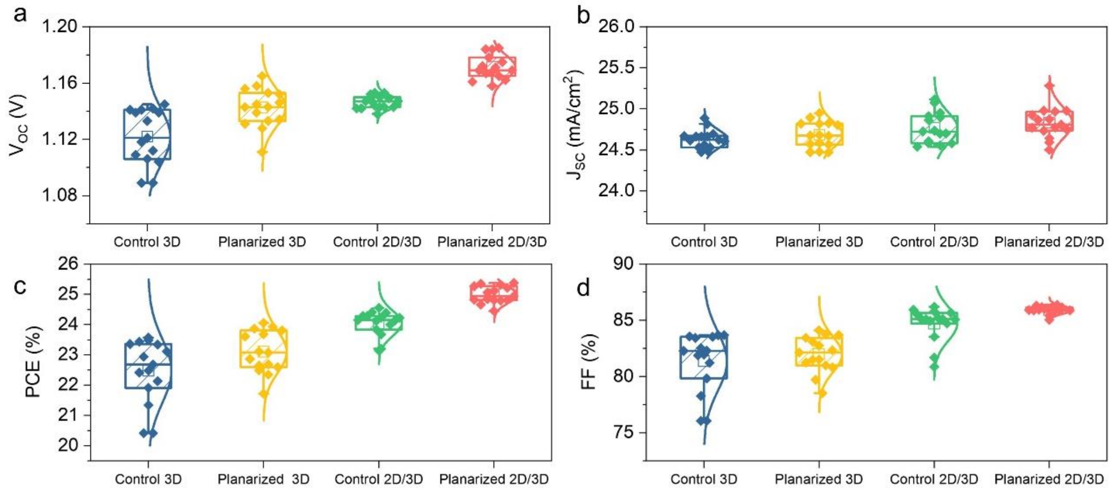

Figure S22. Device performance parameter values include (a) V_{OC}, (b) J_{SC}, (c) PCE, and (d) FF for the control 3D, planarized 3D, control 2D/3D and planarized 2D/3D PSCs.

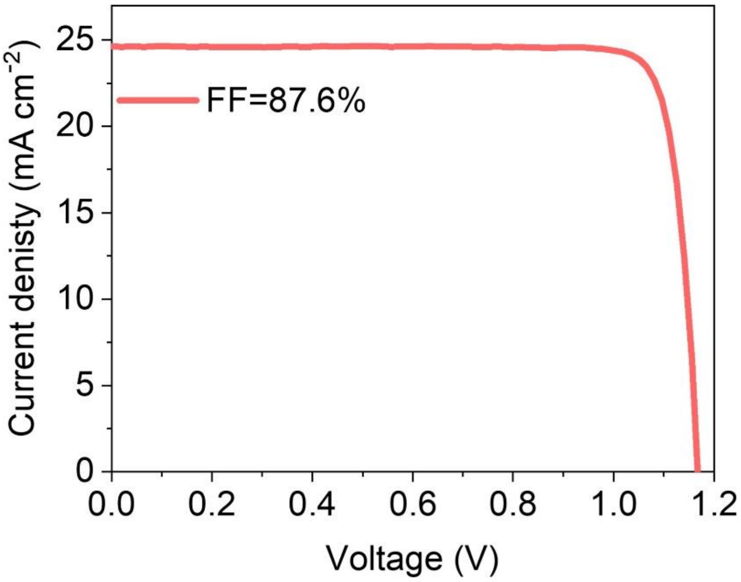

Figure S23. The J-V curve of the planarized 2D/3D perovskite shows a record FF of 87.6%.

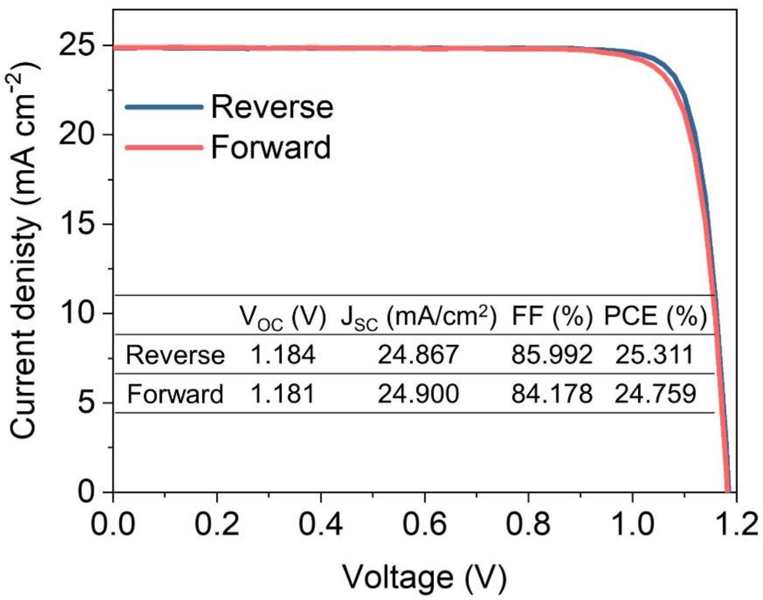

Figure S24. The reverse and forward J-V curves of the champion planarized 2D/3D PSC.

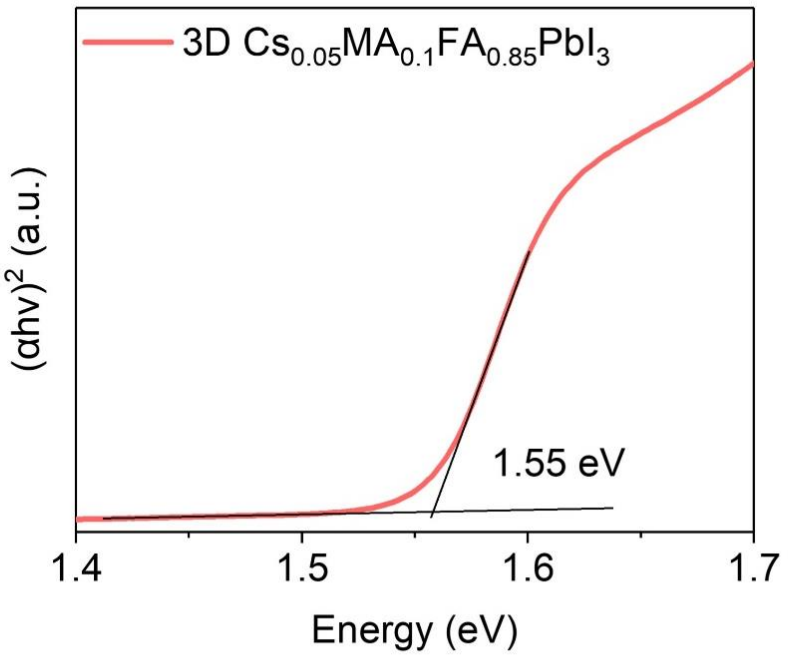

Figure S25. Tauc plot curve of the 3D $Cs_{0.05}MA_{0.1}FA_{0.85}PbI_3$ perovskite.

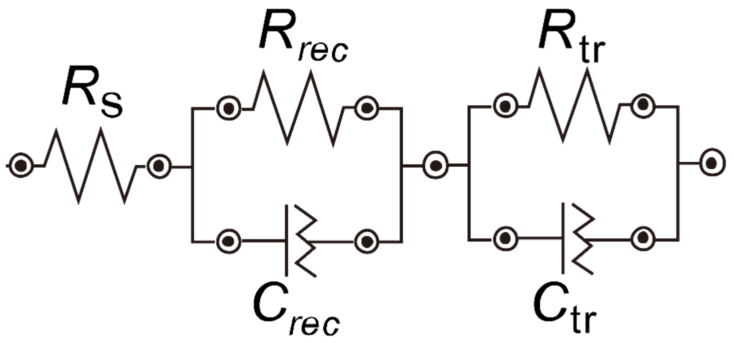

Figure S26. The equivalent circuit used for fitting the Nyquist plots in **Figure 4e**.

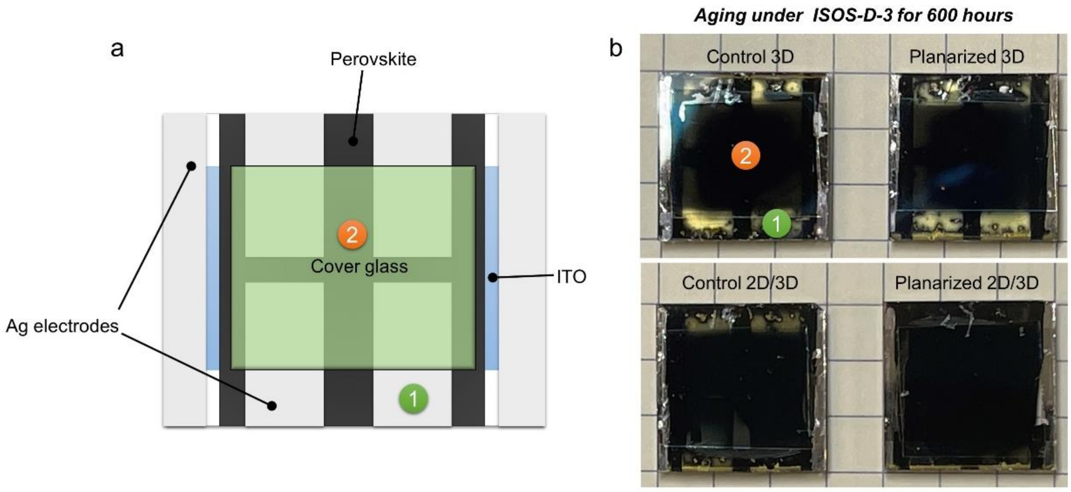

Figure S27. (a) The diagram of the encapsulated device. (b) The optical images of the devices after aging under ISOS-D-3 for 600 hours.

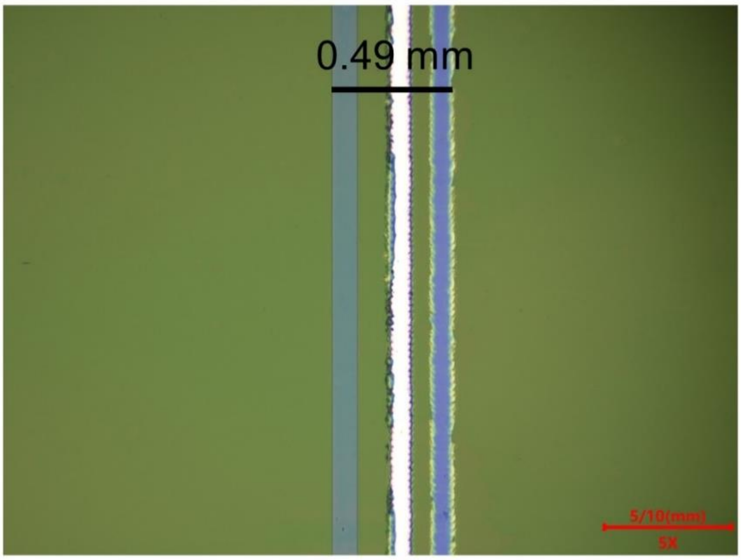

Figure S28. The laser patterning of the P1, P2, and P3 lines of the perovskite solar module.

福建省计量科学研究院
FUJIAN METROLOGY INSTITUTE
（国家光伏产业计量测试中心）
National PV Industry Measurement and Testing Center

检 测 报 告
Test Report

报告编号：24Q3-00301
Report No.

客户信息 Name of Custome	Longbin Qiu Group, Southern University of Science and Technology
联络信息 Contact Information	1088 Xueyuan Avenue, Nanshan District, Shenzhen 518055, China
物品名称 Name of items	Inverted 3D/2D perovskite solar module(IV)
型号/规格 Type /Specification	6cm×6cm
物品编号 Items No	M1
制造厂商 Manufacturer	Longbin Qiu Group, Southern University of Science and Technology
物品接收日期 Items Receipt Date	2024-05-22
检测日期 Test Date	2024-05-23

批准人：Approved by 黎健生
核验员：Checked by 何翔
检测员：Test by 陈彩云

福建省计量科学研究院 证书/报告专用章 3501020056403

发布日期：2024 年 05 月 29 日
Date of Report Year Month Day

扫一扫 查真伪

本院/本中心地址：福州市屏东路9-3号 Address: 9-3 Pingdong Road,Fuzhou,China
电话：0591-87845050 Telephone
传真：0591-87808417 Fax
邮编：350003 Post Code
网址：www.fjjl.net Web Site
咨询电话：0591-87845050 Inquire line
投诉电话：0591-87823025 Complaint Tel

未经本院/本中心书面批准，部分采用本报告内容无效。
Partly using this Report will not be admitted unless allowed by FMI Center.

第 1 页/共 6 页
Page of Pages

福建省计量科学研究院
FUJIAN METROLOGY INSTITUTE
（国家光伏产业计量测试中心）
National PV Industry Measurement and Testing Center

24Q3-00301

检测结果/说明：
Results of Test and additional explanation

1. Standard Test Condition (STC): Total Irradiance: 1000 W/m^2
Temperature: 25.0 °C
Spectral Distribution: AM1.5G

2.Measurement Data and I-V/P-V Curves under STC

Forward Scan

I_{sc}(mA)	V_{oc}(V)	I_{MPP}(mA)	V_{MPP}(V)	P_{MPP} (mW)	FF(%)	A (cm^2)
74.74	8.289	66.83	6.859	458.4	73.99	24.04

Reverse Scan

I_{sc}(mA)	V_{oc}(V)	I_{MPP}(mA)	V_{MPP}(V)	P_{MPP} (mW)	FF(%)	A (cm^2)
74.74	8.345	70.87	7.038	498.8	79.97	24.04

Mismatch factor:0.9926

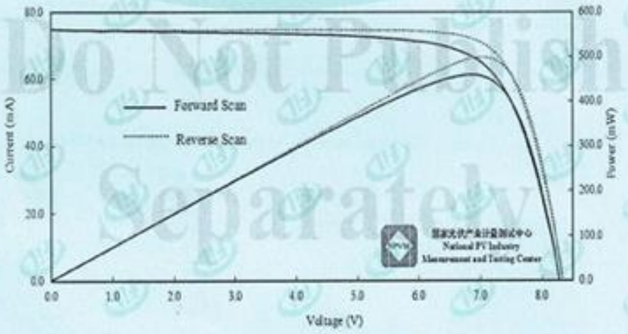

Figure 1. I-V and P-V characteristic curves of the measured sample under STC

检测报告续页专用
Continued page of test report

第 3 页/共 6 页
Page of Pages

Figure S29. Certified efficiency of 20.75% with a designated area of 24.04 cm^2 for planarized 2D/3D PSM in the National Photovoltaic Industry Metrology and Testing Center.

福建省计量科学研究院
FUJIAN METROLOGY INSTITUTE
（国家光伏产业计量测试中心）
National PV Industry Measurement and Testing Center

报告编号： 24Q3-00301
Report No.

检测结果/说明：
Results of Test and additional explanation.

4.Pictures of the Measured Sample

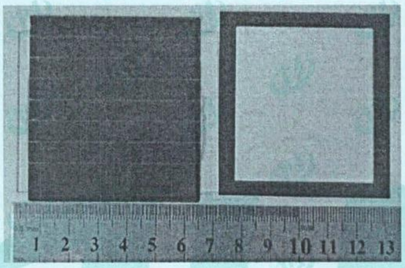

Figure 3. Mask used during test and obverse side of the measured sample

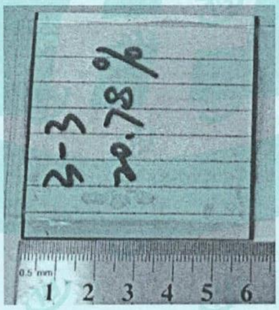

Figure 4. Reverse side of the measured sample

Uncertainty of measurement results:
Short-Circuit Current: U_{rel}=2.5 % (k=2); Open-Circuit Voltage: U_{rel}=1.0 % (k=2);
Maximum Power: U_{rel}=2.2 % (k=2); Efficiency: U_{rel}=2.2 % (k=2); Fill Factor: U_{rel}=3.2 % (k=2).

Explanation：The measured area refers to designated illuminated area.

检测报告续页专用
Continued page of test report

第 5 页/共 6 页
Page of Pages

Figure S30. The optical images of the mask and module. The aperture area of the mask is 24.04 cm^2.

福建省计量科学研究院
FUJIAN METROLOGY INSTITUTE
（国家光伏产业计量测试中心）
National PV Industry Measurement and Testing Center

报告编号： 24Q3-00301
Report No.

检测结果/说明：
Results of Test and additional explanation.

3.Measurement Data and Curves for MPPT under STC

η (%)	20.18
P_{MPP} (mW)	485.1
I_{MPP} (mA)	70.07
V_{MPP} (V)	6.923

Note: Measurement data for MPPT under STC in the above table was the mean value acquired during the final 30 seconds of the 300 seconds test

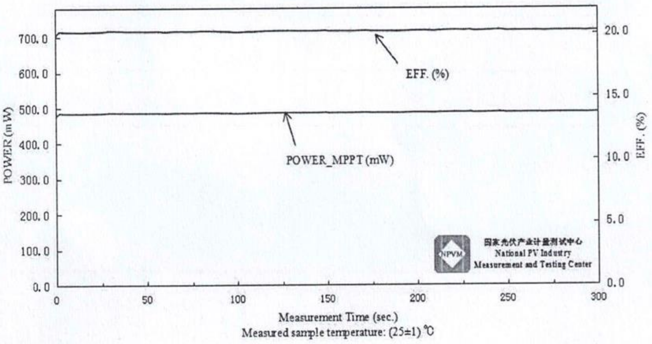

Figure 2. Measurement curves of the measured sample for MPPT

检测报告续页专用
Continued page of test report

第 4 页/共 6 页
Page of Pages

Figure S31. The certified stabilized maximum power point tracking of the module with an efficiency of 20.18%.

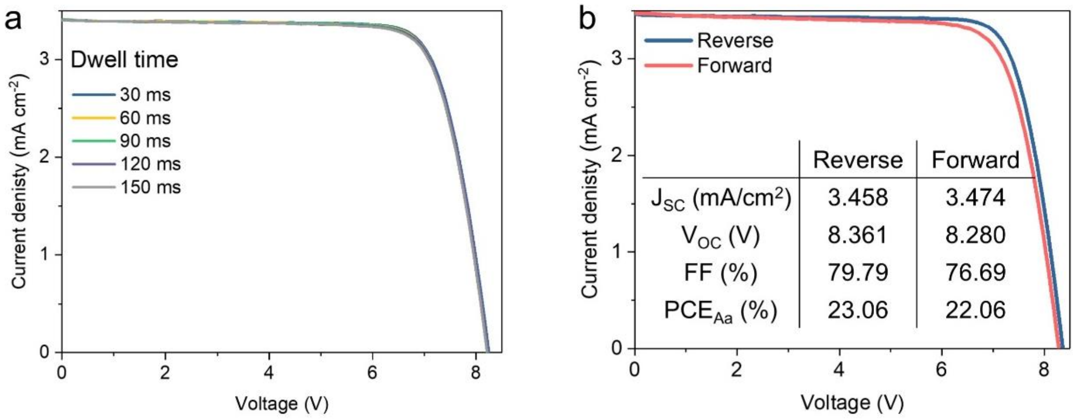

	Reverse	Forward
J_{SC} (mA/cm^2)	3.458	3.474
V_{OC} (V)	8.361	8.280
FF (%)	79.79	76.69
PCE_{Aa} (%)	23.06	22.06

Figure S32. (a) J-V curves of the planarized 2D/3D perovskite solar module under different dwell times. (b) The reverse and forward J-V curves of the champion planarized 2D/3D perovskite solar module.

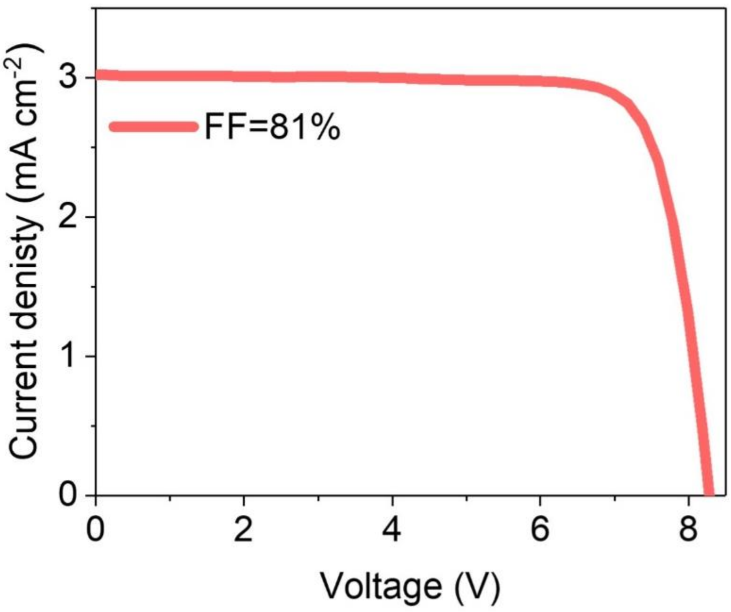

Figure S33. The J-V curve of the planarized 2D/3D perovskite solar module with a champion FF of 81%.

Table S1. Summary of previously reported certified perovskite solar modules.

Device architecture	Area (cm^2)	I_{SC} (mA)	V_{OC} (V)	FF (%)	PCE (%)	Testing Organization	References
p-i-n (PTAA)	29.5^{ap}	83.6	8.27	75.4	18.6	NREL	*Nat. Energy* **6**, 633–641 (2021).
p-i-n (PTAA)	50^{ap}	76.3	16.10	78.0	19.2	NREL	*Science* **373**, 902-907 (2021).
n-i-p	31.0^{ap}	70.1	11.68	73.8	17.53	Newport	*Joule* **5**, 2420–2436 (2021).
n-i-p	17.11^{ap}	64.6	6.44	72.5	17.6	NIM	*Energy Environ. Sci.* ***15**, 244-253 (2022).*
p-i-n (PTAA)	12.84^{ap}	48.9	7.20	80.2	21.07	NPVM	*Adv. Energy Mater*. **12**, 2202287 (2022).
n-i-p	17.11^{ap}	65.1	6.99	73.8	19.6	NIM	*Science* **379**, 288-294 (2023)
p-i-n (PTAA)	26.9^{ap}	76.0	9.38	79.5	21.8	NREL	*Science* **380**, 823-829 (2023).
n-i-p	17.11^{ap}	66.2	6.72	74.4	19.3	NIM	*Nat. Energy* **8**, 294–303 (2023).
p-i-n (PTAA)	79.67^{ap}	--	--	--	19.6	NREL	*Nat Commun* **15**, 1355 (2024).
n-i-p	31^{ap}	83.2	10.03	79.7	21.55	NPVM	*Nat. Energy* **9**, 316–323 (2024).
n-i-p	27.22^{ap}	84.0	9.40	79.2	23.30	NPVM	*Nature* **628**, 299–305 (2024).
**p-i-n (SAMs)**	24.04^{da}	74.7	8.35	**80.0**	20.75	NPVM	**This work**

(Note: ap, aperture area; da, designated area; NREL, National Renewable Energy Laboratory; NIM, National Institute of Metrology; NPVM, National PV Industry Measurement and Testing Center)

Table S2. The contact angle and calculated surface energy of the control and planarized 3D perovskites using Diiodomethane and Glycerol.

	Diiodomethane Contact Angle (CA) (Degree)	Glycerol CA (Degree)	Surface Energy (mJ m^{-2})		
			Dispersive Component	Polar Component	Total
Control 3D	26.9	66.2	45.45	2.62	48.07
Planarized 3D	20.6	44.5	47.60	9.61	57.21

Table S3. The detailed photovoltaic parameters for the 1.57 eV and 1.55 eV PSCs.

		V_{OC} (V)	J_{SC} (mA/cm^2)	FF (%)	PCE (%)
1.57 eV	Control 3D	1.141	24.62	83.43	23.43
	Planarized 3D	1.153	24.82	82.75	23.68
	Control-2D/3D	1.150	24.90	85.77	24.55
	Planarized 2D/3D	1.184	24.87	85.99	25.31
1.55 eV	Planarized 2D/3D Reverse	1.190	25.62	85.36	26.02
	Planarized 2D/3D Forward	1.187	25.62	84.82	25.79
